# Comparative efficacy of acupuncture-related therapy for migraine: A systematic review and network meta-analysis

**DOI:** 10.3389/fneur.2022.1010410

**Published:** 2022-10-26

**Authors:** Yanjuan Song, Tong Li, Chunlian Ma, Hui Liu, Fengxia Liang, Yi Yang

**Affiliations:** ^1^Graduate School, Wuhan Sports University, Wuhan, China; ^2^College of Acupuncture and Orthopedics, Hubei University of Chinese Medicine, Wuhan, China; ^3^Fitness Monitoring and Chronic Disease Intervention Research Center, Wuhan Sports University, Wuhan, China; ^4^College of Health Science, Wuhan Sports University, Wuhan, China; ^5^Hubei Key Laboratory of Exercise Training and Monitoring, Wuhan Sports University, Wuhan, China

**Keywords:** migraine, acupuncture, network meta-analysis, traditional Chinese medicine, analgesia

## Abstract

**Background:**

Migraine is a worldwide disabling chronic brain disorder, some studies suggest acupuncture-related therapy plays an important role in raising efficiency rates and reducing migraine attacks. However, clinical trials comparing the efficacy of different interventions for migraine are limited and controversial. This network meta-analysis (NMA) was performed to review all randomized controlled trials (RCTs) comparing the effects of acupuncture-related therapy for migraine.

**Methods:**

Randomized controlled trials (RCTs) of acupuncture-related therapy for migraine were searched in the following databases from the date of database inception to March 31, 2022, including PubMed, Embase, Cochrane Library, Web of Science, China National Knowledge Infrastructure (CNKI), VIP Database, Wanfang Database, and Chinese Biomedical Database (CBM). The primary endpoint was visual analog scale (VAS) scores. The secondary endpoints were the number of migraine days, duration of migraine, and frequency of migraine attacks. We used Cochrane risk of bias to assess the quality of evidence for outcomes.

**Results:**

Thirty-nine studies involving 4379 patients with 13 different acupuncture-related methods were evaluated. According to surface under the cumulative ranking curve value, acupoint injection was ranked the highest (98.0%) in VAS scores, followed by acupoint implantation (79.0%); electroacupuncture was the optimal intervention method (82.4%) in the number of migraine days, followed by embedding needle therapy (73.1%); embedding needle therapy ranked first (99.9%) in the duration of migraine, followed by acupoint injection (77.4%); acupoint injection was the best intervention (99.3%) in the frequency of migraine attacks, followed by conventional acupuncture plus massage (73.8%).

**Conclusion:**

These results provide preliminary evidence that acupuncture-related therapy could be recommended as one of the effective treatments for migraine. Conventional acupuncture has significant effects on improving VAS scores, the number of migraine days, duration of migraine, and frequency of migraine attacks. However, more high-quality studies should be carried out to verify this finding.

**Systematic review registration:**

https://inplasy.com/, identifier: INPLASY202110035.

## Introduction

Migraine is one of the most common disabling neurological disorders, which is characterized by recurrent episodes of moderate to severe headaches, usually pulsatile and unilateral headache, accompanied by emotional and cognitive symptoms (1, 2). The cause of migraine is complex and related to genetic, environmental, and endogenous factors (3). Based on the survey conducted previously, 11.6% of worldwide people have been plagued by migraine, 10.1% in Asia, and Europe and North America each accounted for 11.4 and 9.7% (4). With the increasing prevalence, the lives and work of migraine sufferers have been severely affected, imposing a tremendous burden on the overall health of the world's people, quality of life, and social medical security system, which also makes it the second most important cause of disability in the world (5–7).

At present, preventive drugs and analgesics still serve as a major therapy choice for migraine, such as beta-blockers, calcium channel blockers, and angiotensin II receptor antagonists (8, 9). However, recurrent migraine requires long-term drug therapy, which may lead to acute drug overuse, drug addiction, and drug dependence in some patients, resulting in a wide range of serious adverse effects (10, 11). Several studies have reported that migraine is a highly burdensome disease (12, 13), including increased heavy economic pressure and psychological burdens such as depression, anxiety, and tension, which seriously affect the personal life and treatment progress of migraine patients (14).

Therefore, there is an urgent need for a safe and effective alternative therapy to improve the symptoms of migraine patients (15). Compared with drug therapy, acupuncture is increasingly used in the integrative and complementary treatment of pain diseases due to its long-term effectiveness, good tolerance, and fewer side effects (16, 17). In the context of what has been hitherto known from the literature, acupuncture has advantages in reducing the frequency and days of migraine attacks and relieving pain severity so that the use of drug therapy will be decreased, and the cost of acupuncture to prevent migraine will also be reduced, which will relieve the economic pressure on society and patients (18–20). Nevertheless, the use of acupuncture and related therapies remains controversial, the published pair-wise meta-analysis shows only the results of direct comparisons, so indirect comparison is needed to provide evidence support for which type of acupuncture therapy may be the most effective method.

To solve the uncertainties described above, we have systematically collected the evidence of acupuncture in the treatment of migraine, then use a comprehensive systematic review and network meta-analysis to conduct direct and indirect comparisons. Besides, the above analysis results can also generate a ranking of different acupuncture therapies based on efficacy so as to assess the effectiveness and safety of acupuncture-related therapy and help physicians make treatment strategies for migraine patients (21, 22).

## Methods

The network meta-analysis research protocol was registered at the INPLASY under the code INPLASY202110035 (Registration doi: 10.37766/ inplasy2021.1.0035). The present analysis was conducted following the Preferred Reporting Items for Systematic Reviews and Meta-Analyses for Network Meta-Analysis (PRISMA-NMA) checklist (23) (Additional file 1).

### Data sources and search strategy

We performed a comprehensive and systematic search from initiation until March 31, 2022, based on the databases of PubMed, Embase, Cochrane Library, Web of Science, China National Knowledge Infrastructure (CNKI), VIP Database, Wanfang Database, and Chinese Biomedical Database (CBM) to identify eligible randomized controlled trials (RCTs), without any restriction on language. The following keywords combined with Medical Subject Headings (MeSH) terms were used for searching: “acupuncture” “acupuncture Therapy”, “acupuncture analgesia”, “migraine”, “disorder, migraine”, “randomized controlled trial”, and “random allocation”. The details of the search strategy are shown in Additional file 2.

### Study selection

Two independent reviewers (YS and TL) screened all inclusion trials to exclude irrelevant studies based on the titles and abstracts of the cited citations with EndNote X 20.0 software. Subsequently, qualified research would be identified by full-text scanning by two researchers. For duplicate citation studies, the latest published RCTs should be selected for data extraction. In case of a dispute, discrepancies were resolved by consensus of discussion between two reviewers; if any disagreement arises, the final adjudication would be done by a third reviewer (CM).

### Inclusion criteria

For the network meta-analysis, studies that met the following criteria were included: (1) Types of study: studies must be designed as randomized controlled trials (RCTs) acupuncture for migraine, and standard-compliant studies have no language-wide restrictions; (2) Types of patients: patients were diagnosed as migraine with aura, without aura, or other special types, and there were no limitations to gender, age, and the course of the disease, but the diagnostic criteria, inclusion and exclusion criteria must be clear; (3) Types of interventions: patients received acupuncture-related therapy (includes conventional acupuncture, embedding needle therapy, acupressure, electroacupuncture; acupuncture and massage, acupuncture and cupping) in the experimental group, placebo and analgesic (flunarizine hydrochloride, valproic acid, topiramate, gabapentin, sodium valproate, ergotamine tartrate, caffeine, metoprolol, ibuprofen) were employed in the control group; (4) Types of outcomes: one of the following efficacy outcomes and safety endpoints must be reported, including VAS scores, the number of migraine days, duration of migraine, frequency of migraine attacks, and adverse events.

### Exclusion criteria

The following were excluded: (1) duplicate studies; (2) non-randomized controlled trials (RCTs); (3) literature review, animal experiments, conference papers, case reports, systematic reviews, and meta-analyses; (4) studies with unclear results or inconsistent outcomes; (5) the patient's migraine was caused by secondary causes such as cerebral hemorrhage, cerebral thrombosis, hypertension, and arteriosclerosis.

### Data extraction

Two reviewers (HL and YS) independently extracted data from the included RCTs and collected the following information: (1) study characteristics of included RCTs (author name, year of publication, country, age and gender of patients, sample size, type of intervention, report of adverse events, and types of outcome); (2) specific information of acupuncture and related interventions (style of acupuncture, names of acupuncture points used, retention time, frequency, and duration); (3) outcomes (VAS scores, the number of migraine days, duration of migraine, and frequency of migraine attacks). Changes in baseline and post-treatment outcome differences are not indicated, and only the individual means and standard deviations above are indicated, it is recommended to estimate the difference from baseline using the formula in the Cochrane Handbook for Systematic Reviews of Interventions (version 5.1):


(1) $$\begin{array}{l} {\bar{X}}_{change}={\bar{X}}_{post-treatment}-{\bar{X}}_{baseline} \end{array}$$

(2) $$\begin{array}{l} SD_{change}=\;\sqrt{{\left(SD_{baseline}\right)}^{2}+{\left(SD_{post-treatment}\right)}^{2}-2\times r\times SD_{baseline}\times SD_{post-treatment}}\; \end{array}$$

The *r* in the formula represents a correlation coefficient with a value of 0.5 (24).

### Quality assessment

Two investigators (Y.Y and C.M) independently assessed the risk of bias in the included RCTs using the Cochrane Collaboration Tool (25). The following six aspects were evaluated: (1) random sequence generation; (2) allocation concealment; (3) blinding of participants and personnel; (4) blinding of outcome assessment; (5) incomplete outcome data; and (6) selective reporting. The judgments of the above projects were classified into “high risk”, “low risk”, or “unclear risk”. If there was any disagreement, it would be decided by the third reviewer. Disagreements would be decided by the third reviewer (F.L).

### Statistical analysis

First of all, meta-analysis and statistical analysis were performed by using Review Manager (Version 5.3, Cochrane Collaboration, Oxford, UK). The heterogeneity between RCTs would be determined by the calculation of I-square (I^2^), which meant that the random effects model should be selected for analysis when *I*^2^ > 50%, and the fixed effect model was selected if *I*^2^ < 50% (26). The continuous data in this paper were analyzed using the mean difference (MD) of the 95% confidence intervals (CI), subgroup analysis could be done based on country or acupuncture category if necessary. Then, a forest map was generated to show the relative efficacy of each group of interventions. Next, network meta-analysis was performed in the Bayesian framework by using WinBUGS (version 1.4.3, MRC Biostatistics Unit, Cambridge, UK) to indirectly compare the efficacy of acupuncture-related therapy (27). Moreover, the Brooks–Gelman–Rubin (BGR) method was mainly to assess the model's convergence, and the criterion was that the closer the potential scale reduction factor (PSRF) value was to 1, the better convergence (28, 29).

At last, we performed a meta-analysis of the STATA software (Version 14.0; Stata Corporation, College Station, Texas, USA) and analyzed the continuous results using mean difference (MD) and its 95% confidence intervals (CI), then network graphs were created by using the “mvmeta” command in STATA to describe the type of acupuncture and related therapies in the comparison network (30). Additionally, based on the network meta-analysis, the surface under the cumulative ranking curve (SUCRA) was calculated to explain the ranking probabilities for various treatments of different outcomes, the higher the SUCRA score, the higher the therapy effectiveness ranking (31). The funnel plot method would be used to assess the publication bias of the included literature, the Z value and its corresponding *p* value should be calculated, and *p* < 0.05 was considered to be statistically significant (32).

## Results

### Study search

**Figure 1:**
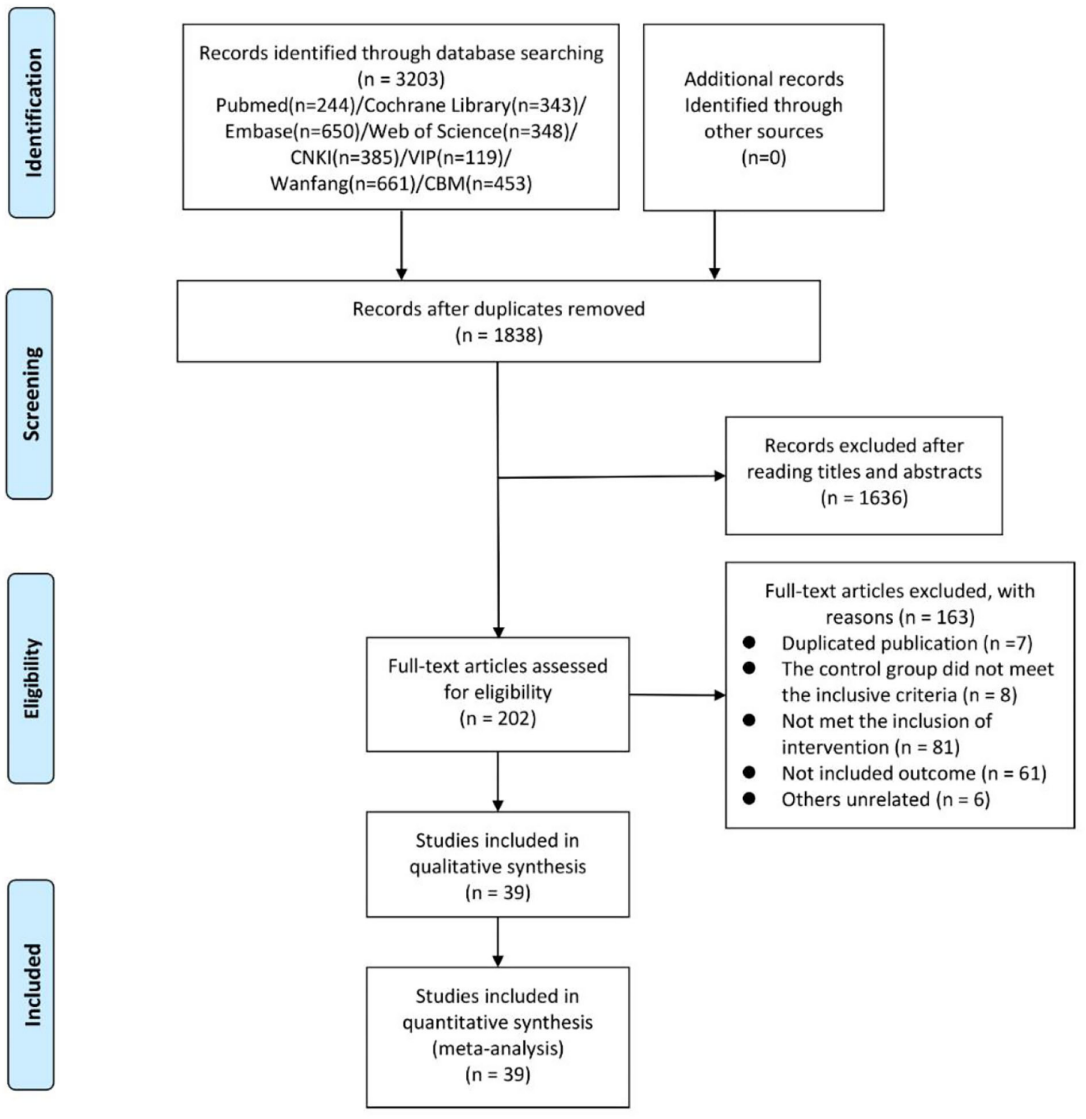
Flow diagram depicting the study selection process (n, number of publications).

The search was performed on March 31, 2022. As illustrated in the flowchart (Figure 1), 3,203 records were identified through electronic search. After duplicates were removed, 1,838 potentially eligible studies were retained in full text. Furthermore, another 1,636 records were excluded by scanning the titles and abstracts. In the remaining 202 articles, full texts were obtained to check eligibility, in which 163 studied were excluded, because the control group and the intervention did not meet the requirements, the outcome indicators were inconsistent, etc. Ultimately, a total of 39 studies with 4,379 participants were involved in our network meta-analysis covering 13 interventions, including conventional acupuncture, massage, analgesic, embedding needle therapy, acupressure, placebo, electroacupuncture, acupuncture cupping, auricular acupuncture, acupoint implantation, acupoint injection, traditional Chinese medicine, and laser acupuncture.

### Study description

**Figure 2:**
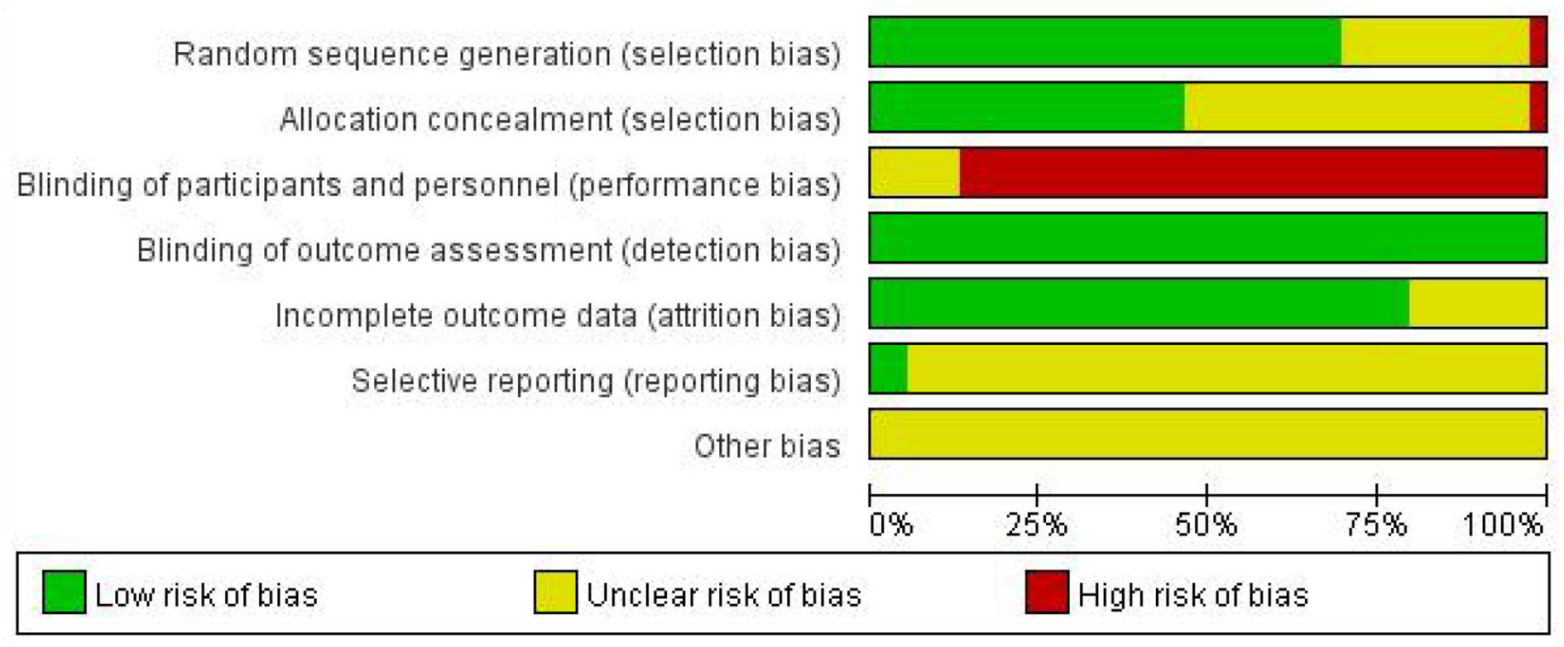
Risk of bias graph.

**Figure 3:**
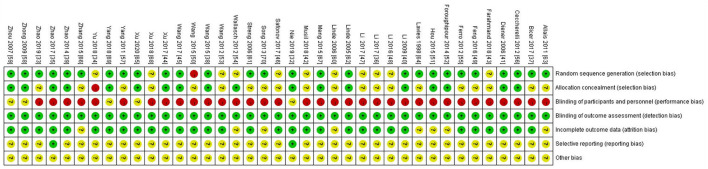
Risk of Bias summary.

**Figure 4:**
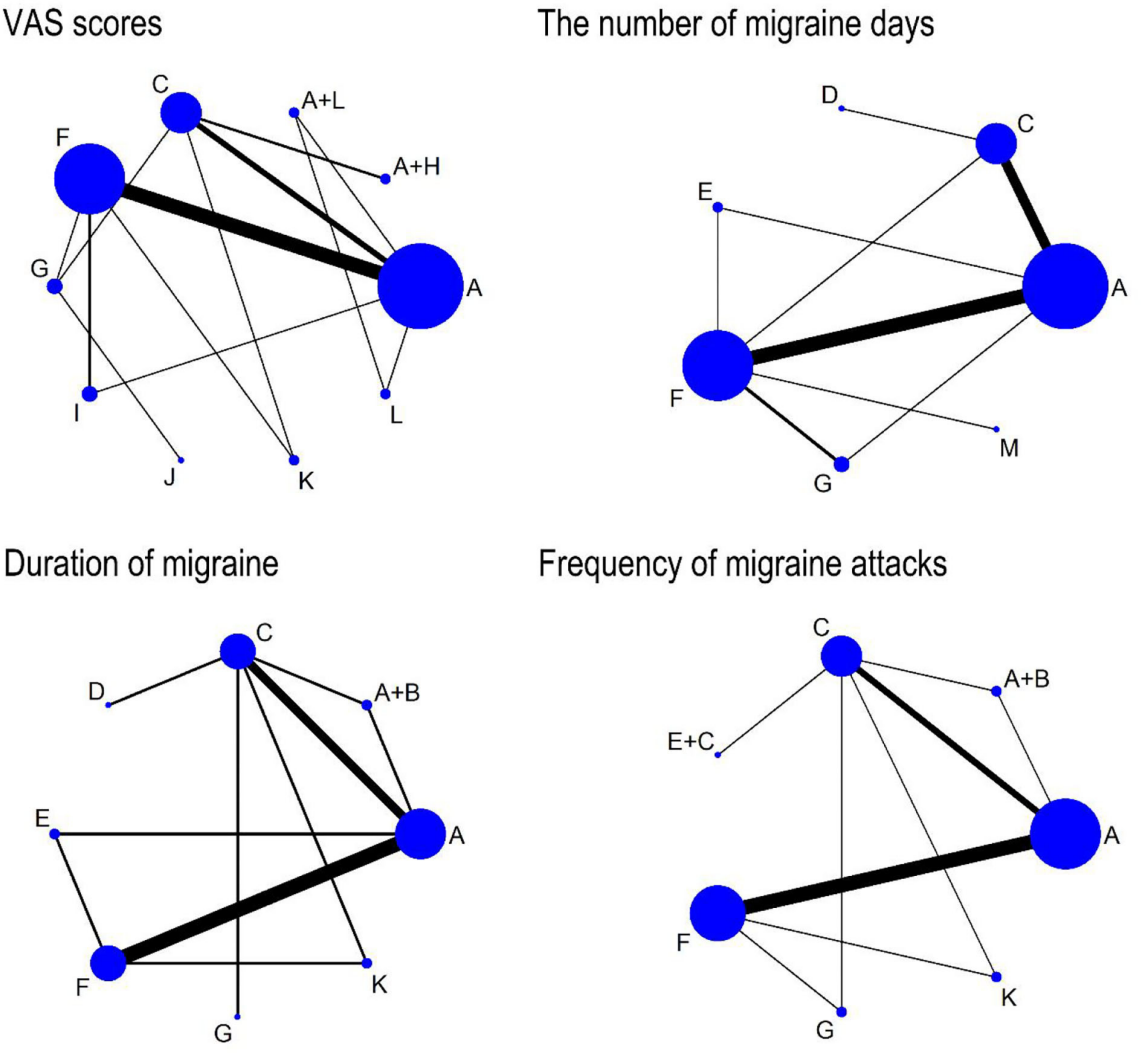
The network comparisons for the outcome of VAS scores, the number of migraine days, duration of migraine, and frequency of migraine attacks. Each node represents an intervention and the size of each node represents the number of randomly assigned participants. Each line represents a direct comparison between interventions and the width of the lines represents the number of studies. A, conventional acupuncture; B, massage; C, analgesic; D, embedding needle therapy; E, acupressure; F, placebo; G, electroacupuncture; H, cupping; I, auricular acupuncture; J, acupoint implantation; K, acupoint injection; L, traditional Chinese medicine; M, laser acupuncture.

Details of the main characteristics incorporated into RCTs are listed in Table 1. Participants ranged in age from 15 to 65 years and the sample size ranged from 5 to 339. The participants were from Canada (35), Turkey (38), Australia (39), Germany (42, 55, 61–63), Czech Republic (43), Iran (44, 53), Russia (47), Brazil (56), Padova (57), Italy (64), Britain (65), and China (36, 40, 41, 48–52, 54, 58–60). All the included studies were designed as RCTs, there were 34 two-arm trials and 5 three-arm trials. Additionally, there were 22 studies comparing the efficacy of acupuncture with placebo and 15 studies comparing the efficacy of acupuncture with an analgesic. Two studies compared combined therapies to acupuncture alone. Specific information about the style of acupuncture, names of acupuncture points used, retention time, and frequency and duration of acupuncture sessions are shown in Table 2. Among the studies, 25 articles (39–41, 48–52, 54, 56, 57, 60, 64) reported VAS scores, 17 articles (34–36, 38–40, 42, 43, 47, 55, 58, 61–63, 65, 66, 70) reported the change in the number of migraine days, 12 articles (33–35, 38, 39, 43, 46, 51, 52, 55, 59, 68) reported duration of migraine, while 18 articles (36, 40, 45, 48, 49, 51–53, 59, 61–63) reported frequency of migraine attacks. Detailed information on the mean, standard deviation (SD), and sample size between the different treatment groups applicable to studies is summarized in Table 3. The Cochrane risk of bias assessment is shown in Figures 2, 3. Besides, the network plot comparing the relationship between acupuncture and related therapies is presented in Figure 4.

**Table 1:** Main characteristics of the included studies.

**Study ID and Country**	**Sample size (Treatment Group/ Control Group)**	**Age: mean (SD) or range (Treatment Group/ Control Group)**	**Interventions**		**Adverse events reported (Treatment Group/ Control Group)**	**Outcomes**
			**Treatment Group**	**Control Group**		
Nie et al. (33), China	45/45	38.2 (5.1)/ 38.9 (5.0)	A+B	
	45/45	39.1 (4.8)/ 38.9 (5.0)	A	C	None	③, ④
Zhao et al. (34), China	18/18	35.6 (4.9)/ 36.6 (7.6)	D	C	None/lassitude, emotional change, weight gaining	②, ③
Yu and Salmoni (35), Canada	7/5	22–52	A	F	NR	②, ③
	6/5		E			
Zhao et al. (36), China	83/80	36.4 (14.2)/ 39.1 (14.6)	G	F	Tingling sensation (3), swelling of the left ankle (1), subcutaneous hemorrhage (1)/Subcutaneous hemorrhage (1)	①, ②, ④
Li et al. (37), China	32/32	37.3 (9.5)/ 36.9 (9.3)	A+H	C	NR	①
Bicer et al. (38), Turkey	29/25	33.76 (7.82)/ 32.0 (9.1)	A	C	NR	①, ②, ③, ④
Wang et al. (39), Australia	26/24	41.6 (14.9)/ 43.8 (13.4)	A	F	Dizziness (4), Bruising (3), Pain (3), Cold and sweaty (8), Tingling (11), Recurrent headache (7), Mild spasm in the calf muscle (1)/Dizziness (3), Bruising (1), Pain (2), Cold and sweaty (5), Tingling (1), Recurrent headache (2)	②, ③
Zhao et al. (40), China	40/40	33.35 (11.69)/ 33.23 (9.73)	A	F	dizziness and sweating (1), minor hemorrhage (2)/ minor hemorrhage (2)	②, ④
Li et al. (41), China	56/56	41.84 (14.21)/ 39.65 (12.83)	A	F	Fainted (3), nausea and vomiting (1), mild bleeding and hematoma (4), pain and distention (1)	
Diener et al. (42), Germany	313/339	37.1 (10.5)/ 38.3 (10.4)	A	F	serious adverse events (5)/serious adverse events (5)/serious adverse events (1)	②
	313/308	37.1 (10.5)/ 36.8 (10.4)		C		
Musil et al. (43), Czech Republic	42/44	45.6 (12.8)/ 46.5 (10.3)	A	C	Facial hematoma (1)	①, ②, ③
Farahmand et al. (44), Iran	30/30	18–65	I	F	NR	
Xu and Mi (45), China	49/49	38.4 (10.7)/ 39.2 (11.3)	E+C	C	NR	④
Wang et al. (46), China	19/19	30 (6)/ 31 (7)	A	F	NR	③, ④
Safonov and Naprienko (47), Russia	22/20	18–63	A	F	NR	②
	20/20		G			
Li et al. (48), China	12/13	21.75 (1.65)/ 21.38 (1.05)	A	F	NR	④
Li et al. (49), China	13/11	21.00 (1.68)/ 21.18 (0.98)	A	F	NR	④
Feng (50), China	30/30	45 (8)/ 44 (8)	J	G	None	①
Wang et al. (51), China	32/32	31 (10)/ 33 (9)	K	C	NR	①, ③, ④
Hou et al. (52), China	42/19	41.0 (9.1)/ 41.7 (8.8)	K	F	Ethereal pain in local skin (1)/ None	①, ③, ④
1 Foroughipour et al. (53), Iran	50/50	35.8 (10.9)/ 37.2 (11.2)	A	F	NR	④
Wang et al. (54), China	75/75	37.8 (10.6)/ 38.6 (12.6)	A	F	Bleeding in a few acupoints (3)/ bleeding in a few acupoints (2), fatigue (2)	①
Wallasch et al. (55), Germany	18/17	37.2 (9.6)/ 39.3 (11.7)	A	F	NR	③
Ferro et al. (56), Brazil	22/23	40.6 (9.1)/ 37.3 (8.6)	A+L	L	NR	①
	22/23	38.2 (7.4)/ 37.3 (8.6)	A			
Ceccherelli et al. (57), Padova	18/17	42.1 (4.4)/ 41.8 (4.6)	A	I	NR	①
Yang et al. (58), China	33/33	48.3 (6.3)/ 48.3 (7.1)	A	C	None/somnolence (6) and nausea (5).	②
Zhong et al. (59), China	114/104	29.7 (5.2)/ 30.2 (4.5)	G	C	None/somnolence (1), hypodynamia (1), mild edema (1)	③, ④
Zhou et al. (60), China	146/140	41.9 (12.3)/ 43.1 (12.7)	G	C	None/somnolence, red complexion	①
Linde et al. (61), Germany	145/81	43 (29–59)	A	F	NR	②, ④
Streng et al. (62), Germany	59/55	40.0 (11.4)/ 40.3 (10.7)	A	C	Mild adverse therapy effects	②, ④
Linde et al. (63), Germany	145/81	43.3 (11.8)/ 41.3 (10.2)	A	F	Fatigue (6), hematoma (4)/ Fatigue (1), hematoma (2)	②, ④
Allais et al. (64), Italy	46/48	15–60/ 16–58	I	F	NR	①
Lavies (65), Britain	6/6	NR	M	F	NR	②
Xu et al. (66), China	58/60	36.6 (12.0)/ 36.0 (10.9)	A	F	Dermorrhagia (2), Sharp pain (1), Palpitation (2)/ None	①, ②, ④
Zhang et al. (67), China	69/64	43.26 (11.87)/ 43.56 (11.60)	A	C	NR	①
Meng et al. (68), China	30/30	32.65 (12.73)/ 30.09 (13.39)	A	F	NR	①, ③, ④
Xu and Wang (69), China	30/30	45.07 (2.34)/ 50.80 (1.89)	A	F	None	
Yang et al. (70), China	20/19	34.5 (3.3)/ 31.4 (2.8)	A	C	Dermorrhagia (7)/Somnolence (3), Constipation (2), Depression (1), Myalgia (1)	②, ④
Song et al. (71), China	45/45	35.4 (3.1)/ 36.1 (2.3)	A+H	C	None/Fatigue (32), Burning sensation in the stomach (2)	

A, conventional acupuncture; B, massage; C, analgesic; D, embedding needle therapy; E, acupressure; F, placebo; G, electroacupuncture; H, cupping; I, auricular acupuncture; J, acupoint implantation; K, acupoint injection; L, traditional chinese medicine; M, laser acupuncture. NR, not reported; ①, VAS scores; ②, the number of migraine days; ③, duration of migraine; ④, frequency of migraine attacks.

**Table 2:** Descriptions of the included acupuncture and related therapies.

**Study ID (Country)**	**Style of acupuncture**	**Names of acupuncture points used**	**Retention time**	**Frequency and duration of acupuncture sessions**
Nie et al. (33), China	A	Yintang (GV29), Touwei (ST8), Taiyang (EX-HN5), Shuaigu (GB8), Fengchi (GB20), Baihui (GV20), Touqiaoyin (GB11)	30 min	Twice a week in the first 4 weeks, once a week during weeks 5–8, and once every 14 days during weeks 9–12
Zhao et al. (34), China	D	Sizhuong (TE23), Hanyan (GB4), Wangu (GB12), Cuanzhu (BL2)	24 h	3 sessions weekly for 4 weeks
Yu and Salmoni (35), Canada	A	Taichong (LR3), Hegu (LI4), Sanyinjiao (SP6), Fengchi (GB20)	20 min	3 sessions monthly with a total of 9 sessions for 3 weeks
	E	Taichong (LR3), Hegu (LI4), Sanyinjiao (SP6), Fengchi (GB20)	15 min	3 sessions monthly with a total of 9 sessions for 3 weeks
	F	Xiguan (LR7), Yangjiao (GB35), Zhouliao (LI12), Dingchuan (EX-B1)	20 min	3 sessions monthly with a total of 9 sessions for 3 weeks
Zhao et al. (36), China	G	Fengchi (GB20), Shuaigu (GB8), Waiguan (SJ5), Kunlun (BL60), Yanglingquan (GB34), Houxi (SI3), Hegu (LI4), Neiting (ST44), Taichong (LR3), Qiuxu (GB40)	30 min	5 sessions weekly with a total of 20 sessions for 4 weeks
	F	Four non-points	30 min	5 sessions weekly with a total of 20 sessions for 4 weeks
Li et al. (37), China	A	Sizhukong (TE23), Shuaigu (GB8), Waiguan (TE5), Zulinqi (GB41), Sizhukong (TE23), Shuaigu (GB8), Waiguan (TE5), Zulinqi (GB41)	20 min	5 sessions weekly with a total of 10 sessions for 2 weeks
Bicer et al. (38), Turkey	A	Baihui (GV20), Yangbai (GB14), Cuanzhu (BL2), Tianzhu (BL10), Yintang (GV29), Hegu (LI4), Quchi (LI11), Zusanli (ST36), Taichong (LR3)	30 min	Three times a week in the first 4 weeks, Twice a week during weeks 5–7
Wang et al. (39), Australia	A	Fengchi (GB20), Shuai Gu (GB8), Taiyang (EX-HN5), Hegu (LI4)	25 min	Twice a week in the first 4 weeks, once a week during weeks 5–8,then once every two weeks during weeks 9–12,once per month in the last two months
	F	1–2 cm away from the real acupoints	25 min	Twice a week in the first 4 weeks, once a week during weeks 5–8,then once every two weeks during weeks 9–12,once per month in the last two months
Zhao et al. (40), China	A	Waiguan (SJ5), Fengchi (GB20), Yanglingquan (GB34), Qiuxu (GB40), Erheliao (SJ22), Daling (PC7), Guangming (GB37), Taibai (SP3)	30 min	4 sessions weekly with a total of 32 sessions for 8 weeks
	F	Inactive acupoints which were chosen according to their anatomical locations, corresponding to Chinese meridians	30 min	4 sessions weekly with a total of 32 sessions for 8 weeks
Li et al. (41), China	A	Waiguan (TE5), Yanglingquan (GB34), Qiuxu (GB40), Jiaosun (TE20), Fengchi (GB20)	30 min	1 session weekly for 1 week
	F	Non-acupoints	30 min	1 session weekly for 1 week
Diener et al. (42), Germany	A	NR	30 min	2 sessions weekly with a total of 12 sessions for 6 weeks
	F	NR	30 min	2 sessions weekly with a total of 12 sessions for 6 weeks
Musil et al. (43), Czech Republic	A	Fengchi (GB20), Shuaigu (GB8), Taiyang (EX-HN5), Baihui (DU20), Xingjian (LR2), Taichong (LR3), Taixi (KI3), Xuanzhong (GB39), Sanyinjiao (SP6), Hegu (LI4), Baihui (DU20), Shangxing (DU23), Zusanli (ST36), Sanyinjiao (SP6), Fenglong (ST40), Zhongwan (CV12), Yinlingquan (SP9), Sanyinjiao (SP6), Xuehai (SP10), Ashi point	25 min	Twice a week in the first 4 weeks, once a week during weeks 5–8, and once every 14 days during weeks 9–12
Farahmand et al. (44), Iran	I	NR	24 h	only one treatment, the treatment time is 4 h
	F	NR	24 h	only one treatment, the treatment time is 4 h
Xu and Mi (45), China	E	Baihui (GV20), Fengchi (GB20), Taiyang (EX-HN5), Neiguan (PC6)	5 min	3 sessions weekly for 8 weeks
Wang et al. (46), China	A	Toutong point	NR	5 sessions weekly with a total of 20 sessions for 4 weeks
Safonov and Naprienko (47), Russia	A	NR	NR	2 sessions weekly with a total of 8 sessions for 4 weeks
	G	NR	NR	2 sessions weekly with a total of 8 sessions for 4 weeks
Li et al. (48), China	A	Yanglingquan (GB34), Qiuxu (GB40), Waiguan (TE5)	30 min	5 sessions weekly with a total of 20 sessions for 4 weeks
	F	NAP1, NAP2, NAP3	30 min	5 sessions weekly with a total of 20 sessions for 4 weeks
Li et al. (49), China	A	Zusanli (ST36), Chongyang (ST42), Pianli (L16)	30 min	5 sessions weekly with a total of 20 sessions for 4 weeks
	F	NAP1, NAP2, NAP3	30 min	5 sessions weekly with a total of 20 sessions for 4 weeks
Feng (50), China	J	Fengchi (GB20), Taiyang (EX-HN5), Waiguan (TE5), Yanglingquan (GB34)	NR	2 sessions every two weeks for 4 weeks
	G	Fengchi (GB20), Taiyang (EX-HN5), Yanglingquan (GB34), Zusanli (ST36)	30 min	4 sessions weekly with a total of 16 sessions for 4 weeks
Wang et al. (51), China	K	Naohu (GV17), Luoque(BL8), Naokong (GB19), Shuaigu (GB8), Baihui (GV20), Chengling (GB18), Yuzhen (BL9), Touwei (ST8)	NR	4 sessions monthly with a total of 12 sessions for 3 months
Hou et al. (52), China	K	Yintang (GV29), Taiyang (EX-HN5), Baihui (GV20), Shuaigu (GB8), Fengchi (GB20), Tianzhu (BL10)	NR	1 session monthly for 4 months
Foroughipour et al. (53), Iran	A	NR	30 min	3 sessions weekly with a total of 12 sessions for 4 weeks
	F	NR	30 min	3 sessions weekly with a total of 12 sessions for 4 weeks
Wang et al. (54), China	A	Baihui (GV20), Shenting (GV24), Touwei (ST8), Shuaigu (GB8), Fengchi (GB20)	30 min	NR
	F	30 acupoints unrelated to headache were selected in the vicinity of elbow and knee joints	30 min	NR
Wallasch et al. (55), Germany	A	Hegu (LI4), Zusanli (ST36), Waiguan (TE5), Zulinqi (GB41), Houxi (SI3), Shenmai (BL62), Baihui (GV20), Fengchi (GB20), Taiyang (EX-HN5), Sizhukong (TE23), Taichong (LR3), Taixi (KI3)	30 min	1 session weekly for 8 weeks
	F	Areas of the skin that were outside a classically described acupuncture point (minimum 1–2 cm beside)	30 min	1 session weekly for 8 weeks
Ferro et al. (56), Brazil	A	Shuaigu (GB8), Yangbai (GB14), Baihui (GV20), Shenmen (HT7), Xingjian (LR2), Hegu (LI4)	30 min	2 sessions weekly with a total of 20 sessions for 10 weeks
Ceccherelli et al. (57), Padova	A	Taichong (LR3), Sanyinjiao (SP6), Waiguan (TE5), Fengchi (GB20), Baihui (GV20), Jiache (ST6), Touwei (ST8)	5 min	1 session weekly for 8 weeks
	I	Aggressiveness point, Lung point, Thalamus point	NR	1 session weekly for 8 weeks
Yang et al. (58), Taiwan	A	Cuanzhu (BL2), Fengchi (GB20), Taiyang (EX-HN5), Yintang (GV29)	30 min	2 sessions weekly with a total of 24 sessions for 12 weeks
Zhong et al. (59), China	G	Taichong (LR3), Yanglingquan (GB34), Fengchi (GB20), Ququan (LR8)	20 min	5 sessions weekly with a total of 20 sessions for 4 weeks
Zhou et al. (60), China	G	Taiyang (EX-HN5)	30 min	5 sessions weekly with a total of 20 sessions for 4 weeks
Linde et al. (61), Germany	A	Fengchi (GB20), Qiuxu (GB40), Zulinqi (GB41), Diwuhui (GB42), Baihui (GV20), Taichong (LR3), Zhongzhu (TE3), Waiguan (TE5), Taiyang (EX-HN5)	30 min	Twice a week in the first 4 weeks, once a week during weeks 5–8
	F	Near Binao (LI14)	30 min	Twice a week in the first 4 weeks, once a week during weeks 5–8
Streng et al. (62), Germany	A	Fengchi (GB20), Qiuxu (GB40), Zulinqi (GB41), Diwuhui (GB42), Baihui (GV20), Taichong (LR3), Zhongzhu (TE3), Waiguan (TE5),Taiyang (EX-HN5)	20–30 min	8–15 sessions for 12 weeks.
Linde et al. (63), Germany	A	Fengchi (GB20), Qiuxu (GB40), Zulinqi (GB41), Diwuhui (GB42), Baihui (GV20), Taichong (LR3), Zhongzhu (TE3), Waiguan (TE5),Taiyang (EX-HN5)	30 min	Twice a week in the first 4 weeks, once a week during weeks 5–8
	F	NR	30 min	Twice a week in the first 4 weeks, once a week during weeks 5–8
Allais et al. (64), Italy	I	Area M and Area S in Chinese auricular maps	24 h	NR
	F	Area M and Area S in Chinese auricular maps	24 h	NR
Lavies (65), Britain	M	Taichong (LR3), Zusanli (ST36), Hegu (LI4), Fengchi (GB20)	NR	1 session weekly for 6 weeks
	F	NR	NR	1 session weekly for 6 weeks
Xu et al. (66), China	A	Hegu (LI4), Taichong (LR3), Taiyang (EX-HN5), Fengchi (GB20), Shuaigu (GB8), Touwei (ST8), Tianzhu (BL10), Baihui (DU20)	30 min	20 sessions acupuncture treatments over 8 weeks.
	F	Sham point 1–2 [Bilateral, at the midpoint between the acupuncture points, Jianjin (GB21) and Jugu (LI16)], Sham point 3–4 (Bilateral, at a distance of 5 cun from the seventh cervical spine), Sham point 5–6 (Bilateral, at a distance of 5 cun from the eighth thoracic spine), Sham point 7–8 (Bilateral, at a distance of 5 cun from the ninth thoracic spine)	30 min	Every other day to fulfill a 10-session treatment course, and another course will begin after resting 9 days
Zhang et al. (67), China	A	Taiyang (EX-HN5), Touwei (ST8), Fengchi (GB20), Hegu (LI4)	NR	1 course of treatment in 10 days, 3 courses in total, 5 days between treatments
Meng et al. (68), China	A	Taiyang (EX-HN5), Shuaigu (GB8), Toulinqi (GB15), Muchuang (GB16), Waiguan (TE5), Zhongzhu (TE3)	60 min	5 sessions weekly with a total of 20 sessions for 4 weeks
	F	Sham point 1(The inner side of the elbow, the midpoint of the line between the tip of the elbow and the armpit), Sham point 2(The midpoint of the line between the inner epicondyle of the humerus and the wrist of the ulna, the ulnar edge), Sham point 3(The junction of the anterior deltoid muscle and biceps in the arm), Sham point 4 (1–2 cm lateral to Zusanli, lateral tibial margin)	60 min	5 sessions weekly with a total of 20 sessions for 4 weeks
Xu and Wang (69), China	A	Taiyang (EX-HN5), Sizhukong (TE23), Jiaosun (TE20), Shuaigu (GB8), Fengchi (GB20), Waiguan (TE5), Zulinqi (GB41)	20 min	2 sessions weekly with a total of 12 sessions for 6 weeks
	F	5-mm outside the acupuncture point	20 min	2 sessions weekly with a total of 12 sessions for 6 weeks
Yang et al. (70), China	A	Shuaigu (GB8), Touwei (ST8), Taiyang (EX-HN5), Fengchi (GB20)	20 min	2–3 sessions weekly for 4 weeks
Song et al. (71), China	A	Mei Hua (Select a group of acupoints along the periphery and midpoint of the pain area), Xiang Leng (The area located within 1.5 cun of both sides of the cervical spine), Taiyang (EX-HN5), Fengchi (GB20), Dazhui (DU14)	NR	2 sessions weekly with a total of 16 sessions for 8 weeks

A, conventional acupuncture; B, massage; C, analgesic; D, embedding needle therapy; E, acupressure; F, placebo; G, electroacupuncture; H, cupping; I, auricular acupuncture; J, acupoint implantation; K, acupoint injection; L, traditional Chinese medicine; M, laser acupuncture; NR, not reported.

**Table 3:** Summarize of mean, standard difference, and sample size between treatment groups for included studies in a network meta-analysis.

**Study**	**Treatment**	**VAS**		**The number**		**Duration of**		**Frequency of**		**N**
		**Scores**		**of migraine days**		**migraine**		**migraine attacks**		
		**Mean**	**SD**	**Mean**	**SD**	**Mean**	**SD**	**Mean**	**SD**	
Nie et al. (33), China	A+B	/	/	/	/	11.11	4.03	2.79	0.9	45
	A	/	/	/	/	9.44	3.6	2.21	0.8	45
	C	/	/	/	/	7.01	3.29	1.4	0.62	45
Zhao et al. (34), China	D	/	/	4.4	3.1	36.3	10.9	/	/	18
	C	/	/	2.1	2.5	14.2	9.9	/	/	18
Yu and Salmoni (35), Canada	A	/	/	1.71	0.68	32.36	23.6	/	/	7
	E	/	/	1.83	0.94	16.58	6.85	/	/	6
	F	/	/	0.4	1.02	5.3	11.44	/	/	5
Zhao et al. (36), China	G	2.1	1.9	3.5	3.3	/	/	2.1	2.3	83
	F	1.4	1.6	2.2	3.8	/	/	3.5	2.4	80
Li et al. (37), China	A+H	4.95	0.89	/	/	/	/	/	/	32
	C	3.64	0.93	/	/	/	/	/	/	32
Bicer et al. (38), Turkey	A	3.35	1.76	4.54	2.11	17.09	15.41	3.24	1.57	29
	C	4.24	2.22	5.08	1.73	14.68	11.66	4.4	1.93	25
Wang et al. (39), Australia	A	1.6	1.6	6.6	5.4	3	3.4	/	/	26
	F	0.8	1.9	2.3	6.7	0.9	4.6	/	/	24
Zhao et al. (40), China	A	2.096	0.25	6.34	6.99	/	/	3.98	3.64	40
	F	1.11	0.25	5.82	6.67	/	/	2.88	3.22	40
Li et al. (41), China	A	1	3.7	/	/	/	/	/	/	56
	F	0.5	1.8	/	/	/	/	/	/	56
Diener et al. (42), Germany	A	/	/	2.3	3.6	/	/	/	/	313
	F	/	/	1.5	3.8	/	/	/	/	339
	C	/	/	2.1	4	/	/	/	/	308
Musil et al. (43), Czech Republic	A	0.18	1.3	5.5	11.2	0.46	5.64	/	/	42
	C	−0.3	0.76	2	4.9	0.07	4.5	/	/	44
Farahmand et al. (44), Iran	I	7.1	1.3	/	/	/	/	/	/	30
	F	6.4	1.9	/	/	/	/	/	/	30
Xu and Mi (45), China	E+C	/	/	/	/	/	/	2	1.2	49
	C	/	/	/	/	/	/	1.9	1.1	49
Wang et al. (46), China	A	2.86	1.32	/	/	11.92	14.01	1.54	0.91	19
	F	0.62	1.45	/	/	3.81	16.26	0.05	1.07	19
Safonov and Naprienko (47), Russia	A	/	/	14.54	1.73	/	/	/	/	22
	G	/	/	14.45	1.74	/	/	/	/	20
	F	/	/	6.4	1.44	/	/	/	/	20
Li et al. (48), China	A	2.08	1.35	/	/	/	/	1.83	2.7	12
	F	1.27	1.48	/	/	/	/	−0.15	3.37	13
Li et al. (49), China	A	2.54	1.22	/	/	/	/	1.46	3.26	13
	F	1.14	1.44	/	/	/	/	0	3.45	11
Feng (50), China	J	5.95	0.38	/	/	/	/	/	/	30
	G	4.67	0.36	/	/	/	/	/	/	30
Wang et al. (51), China	K	5.3	1.5	/	/	40.4	7.9	7.8	1.9	32
	C	2.9	1.6	/	/	36	6.8	5.8	1.6	32
Hou et al. (52), China	K	5	1.3	/	/	6.3	2.3	5.7	1.8	42
	F	0.5	2	/	/	0.2	2.1	0.1	2.4	19
Foroughipour et al. (53), Iran	A	/	/	/	/	/	/	1.7	1.1	50
	F	/	/	/	/	/	/	0.7	0.9	50
Wang et al. (54), China	A	2.4	2.1	/	/	/	/	/	/	75
	F	0.7	2	/	/	/	/	/	/	75
Wallasch et al. (55), Germany	A	/	/	3.73	3	39.9	38.4	/	/	18
	F	/	/	1.23	2.55	15.6	37	/	/	17
Ferro et al. (56), Brazil	A+L	6.4	3.1	/	/	/	/	/	/	22
	A	5.6	2.4	/	/	/	/	/	/	22
	L	3.7	2.1	/	/	/	/	/	/	23
Ceccherelli et al. (57), Padova	A	2.78	1.54	/	/	/	/	/	/	18
	I	2.06	1.87	/	/	/	/	/	/	17
Yang et al. (58), Taiwan	A	/	/	10.5	2.8	/	/	/	/	33
	C	/	/	7.8	3.6	/	/	/	/	33
Zhong et al. (59), China	G	/	/	/	/	3.91	1.16	2.4	0.4	114
	C	/	/	/	/	3.17	1.29	1.8	0.5	104
Zhou et al. (60), China	G	1.4	1.1	/	/	/	/	/	/	146
	C	0.5	1	/	/	/	/	/	/	140
Linde et al. (61), Germany	A	/	/	2.2	2.7	/	/	1.5	1.2	145
	F	/	/	2.2	2.7	/	/	1.6	1.3	81
Streng et al. (62), Germany	A	/	/	2.6	2.7	/	/	1.4	1.4	59
	C	/	/	1.9	3	/	/	1	1.4	55
Linde et al. (63), Germany	A	/	/	3	2.6	/	/	1.3	1.2	145
	F	/	/	2.8	2.5	/	/	1.2	1.2	81
Allais et al. (64), Italy	I	3	0.25	/	/	/	/	/	/	46
	F	0.7	0.23	/	/	/	/	/	/	48
Lavies (65), Britain	M	/	/	−4	3.3	/	/	/	/	6
	F	/	/	−2	6.2	/	/	/	/	6
Xu et al. (66), China	A	2.2	2.5	3.9	3	/	/	2.3	1.7	58
	F	0.9	1.9	2.2	3.2	/	/	1.6	2.5	60
Zhang et al. (67), China	A	3.54	2.03	/	/	/	/	/	/	69
	C	2.92	1.79	/	/	/	/	/	/	64
Meng et al. (68), China	A	2.62	3.07	/	/	1.99	1.12	2.61	1.17	30
	F	2	2.56	/	/	0.64	1.31	0.6	1.42	30
Xu and Wang (69), China	A	1.37	0.29	/	/	/	/	/	/	30
	F	0.47	0.28	/	/	/	/	/	/	30
Yang et al. (70), China	A	3.5	1.45	2.65	1.98	/	/	2.4	1.82	20
	C	1.79	1.28	1.52	1.97	/	/	1.42	1.99	19
Song et al. (71), China	A+H	5.47	1.63	/	/	/	/	/	/	45
	C	3.75	1.66	/	/	/	/	/	/	45

A, conventional acupuncture; B, massage; C, analgesic; D, embedding needle therapy; E, acupressure; F, placebo; G, electroacupuncture; H, cupping; I, auricular acupuncture; J, acupoint implantation; K, acupoint injection; L, traditional Chinese medicine; M, laser acupuncture.

### Pairwise meta-analyses

#### VAS scores

Results from pairwise random-effect meta-analyses showed that conventional acupuncture was superior in improving VAS scores compared with placebo (MD: 1.06, 95% CI, 0.86 to 1.26), while there was no significant difference between conventional acupuncture and analgesic (SMD: 0.36, 95% CI, −0.17 to 0.89). Conventional acupuncture plus cupping was significantly more effective than analgesic (MD: 1.43, 95% CI, 1.06 to 1.81) in reducing VAS scores, while there was no significant difference between conventional acupuncture plus traditional Chinese medicine and conventional acupuncture (MD: 0.80, 95% CI, −0.82 to 2.42). The VAS scores of acupoint implantation were significantly lower than that of electroacupuncture (MD: 1.28, 95% CI, 1.09 to 1.47). VAS scores were significantly lower in conventional acupuncture (MD: 1.90, 95% CI, 0.58 to 3.22) and conventional acupuncture plus traditional Chinese medicine (MD: 2.70, 95% CI, 1.17 to 4.23) than those in traditional Chinese medicine. Acupoint injection (MD: 4.50, 95% CI, 3.52 to 5.48) and electroacupuncture (MD: 0.70, 95% CI, 0.16 to 1.24) were found to have significant effects in reducing VAS scores when compared with placebo. Furthermore, the results showed that acupoint injection (MD: 2.40, 95% CI, 1.64 to 3.16) and electroacupuncture (MD: 0.90, 95% CI, 0.66 to 1.14) were significantly more effective in VAS scores than analgesic. The pooled results also showed that there were no significant differences in VAS scores between auricular acupuncture and placebo (MD: 1.55, 95% CI, −0.01 to 3.12), and conventional acupuncture (MD: 0.72, 95% CI, −0.42 to 1.86) (Table 4).

**Table 4:** Pairwise meta-analyses.

**Comparison**	**MD/SMD(95% CI)**	**Number of patients**	**Number of studies**	***I*^2^ (%)**	***p* value**
VAS scores					
A vs. F	1.06 (0.86 to 1.26)	717	10	44	0.07
A vs. C	0.36 (−0.17 to 0.89)	312	4	79	0.002
A+H vs. C	1.43 (1.06 to 1.81)	154	2	0	0.32
J vs. G	1.28 (1.09 to 1.47)	60	1	-	-
A vs. L	1.90 (0.58 to 3.22)	45	1	-	-
A+L vs. A	0.80 (−0.82 to 2.42)	45	1	-	-
A+L vs. L	2.70 (1.17 to 4.23)	46	1	-	-
I vs. F	1.55 (−0.01 to 3.12)	154	2	93	0.0002
K vs. F	4.50 (3.52 to 5.48)	61	1	-	-
G vs. F	0.70 (0.16 to 1.24)	163	1	-	-
K vs. C	2.40 (1.64 to 3.16)	64	1	-	-
G vs. C	0.90 (0.66 to 1.14)	286	1	-	-
A vs. I	0.72 (−0.42 to 1.86)	35	1	-	-
The number of migraine days					
A vs. F	2.12 (0.35 to 3.90)	1,441	9	96	< 0.00001
A vs. C	0.83 (−0.04 to 1.70)	980	6	69	0.006
A vs. E	−0.12 (−1.03 to 0.79)	13	1	-	-
C vs. F	0.60 (−0.00 to 1.20)	647	1	-	-
M vs. F	−2.00 (−7.62 to 3.62)	12	1	-	-
E vs. F	1.43 (0.26 to 2.60)	11	1	-	-
G vs. F	4.68 (−1.94 to 11.29)	203	2	99	< 0.00001
D vs. C	2.30 (0.46 to 4.14)	36	1	-	-
A vs. G	0.09 (−0.96 to 1.14)	42	1	-	-
Duration of migraine					
A vs. F	0.74 (0.45 to 1.04)	195	5	0	0.49
A vs. C	0.32 (−0.08 to 0.73)	230	3	56	0.10
A vs. E	15.78 (−2.54 to 34.10)	13	1	-	-
A+B vs. C	4.10 (2.58 to 5.62)	90	1	-	-
A+B vs. A	1.67 (0.09 to 3.25)	90	1	-	-
K vs. F	6.10 (4.93 to 7.27)	61	1	-	-
E vs. F	11.28 (−0.15 to 22.71)	11	1	-	-
K vs. C	4.40 (0.79 to 8.01)	64	1	-	-
D vs. C	22.10 (15.30 to 28.90)	36	1	-	-
G vs. C	0.74 (0.41 to 1.07)	218	1	-	-
Frequency of migraine attacks					
A vs. F	0.58 (0.22 to 0.94)	897	9	83	< 0.00001
A vs. C	0.32 (−0.38 to 1.02)	297	4	88	< 0.0001
A+B vs. C	1.39 (1.07 to 1.71)	90	1	-	-
A+B vs. A	0.58 (0.23 to 0.93)	90	1	-	-
E+C vs. C	0.10 (−0.36 to 0.56)	98	1	-	-
K vs. F	5.60 (4.39 to 6.81)	61	1	-	-
G vs. F	−1.40 (−2.12 to −0.68)	163	1	-	-
G vs. C	0.60 (0.48 to 0.72)	218	1	-	-
K vs. C	2.00 (1.14 to 2.86)	64	1	-	-

A, conventional acupuncture; B, massage; C, analgesic; D, embedding needle therapy; E, acupressure; F, placebo; G, electroacupuncture; H, cupping; I, auricular acupuncture; J, acupoint implantation; K, acupoint injection; L, traditional Chinese medicine; M, laser acupuncture.

#### The number of migraine days

With regard to the efficacy of the number of migraine days, the results showed that conventional acupuncture seemed to be better than placebo (MD: 2.12, 95% CI, 0.35 to 3.90). There were no differences in the number of migraine days between conventional acupuncture and analgesic (MD: 0.83, 95% CI, −0.04 to 1.70), acupressure (MD: −0.12, 95% CI, −1.03 to 0.79), and electroacupuncture (MD: 0.09, 95% CI, −0.96 to 1.14). Acupressure was more efficacious than placebo (MD: 1.43, 95% CI, 0.26 to 2.60). Moreover, the number of migraine days of embedding needle therapy was significantly better than those of analgesic (MD: 2.30, 95% CI, 0.46 to 4.14). The remaining direct comparisons showed no significant differences in the effectiveness rate of treatment (Table 4).

#### Duration of migraine

As for the comparison in decreasing the duration of migraine, conventional acupuncture showed a greater reduction than placebo (SMD: 0.74, 95% CI, 0.45 to 1.04), while there were no significant differences in the duration of migraine between conventional acupuncture and analgesic (SMD: 0.32, 95% CI, −0.08 to 0.73) and acupressure (MD: 15.78, 95% CI, −2.54 to 34.10). The pooled results showed that conventional acupuncture plus massage seemed to have a better effect on decreasing the duration of migraine than analgesic (MD: 4.10, 95% CI, 2.58 to 5.62) and conventional acupuncture (MD: 1.67, 95% CI, 0.09 to 3.25) alone. Acupoint injection was superior in improving the duration of migraine compared with placebo (MD: 6.10, 95% CI, 4.93 to 7.27) and analgesic (MD: 4.40, 95% CI, 0.79 to 8.01). Furthermore, embedding needle therapy (MD: 22.10, 95% CI, 15.30 to 28.90) and electroacupuncture (MD: 0.74, 95% CI, 0.41 to 1.07) had a better effect than analgesic. There was no statistically significant difference between acupressure and placebo (MD: 11.28, 95% CI, −0.15 to 22.71) in the duration of migraine (Table 4).

#### Frequency of migraine attacks

This study showed that conventional acupuncture had a better effect than placebo (SMD: 0.58, 95% CI, 0.22 to 0.94) on the reduction of frequency of migraine attacks. Conventional acupuncture plus massage was found to be more effective in the relief of frequency of migraine attacks when they were compared with analgesic (MD: 1.39, 95% CI, 1.07 to 1.71) and conventional acupuncture (MD: 0.58, 95% CI, 0.23 to 0.93). The frequency of migraine attacks acupoint injection (MD: 5.60, 95% CI, 4.39 to 6.81) and electroacupuncture (MD: −1.40, 95% CI, −2.12 to −0.68) was significantly better than that of placebo. The frequency of migraine attacks of electroacupuncture (MD: 0.60, 95% CI, 0.48 to 0.72) and acupoint injection (MD: 2.00, 95% CI, 1.14 to 2.86) was superior than that of analgesic. Moreover, we failed to observe any significant difference regarding the efficacy among other interventions in the frequency of migraine attacks (Table 4).

### Network meta-analysis

#### VAS scores

**Figure 5:**
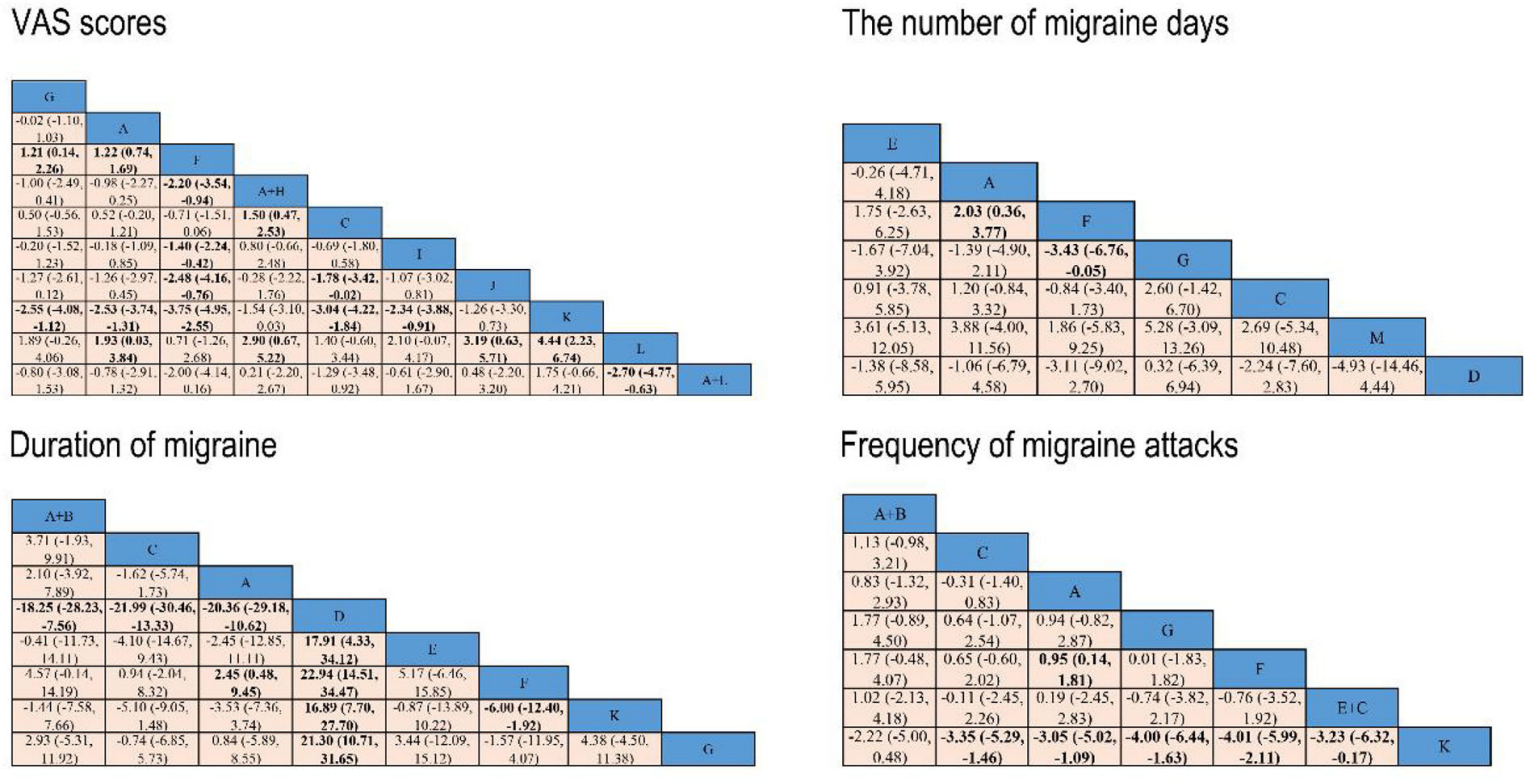
Network meta-analysis results for VAS scores, the number of migraine days, duration of migraine, and frequency of migraine attacks. Significant results are bolded in black. A, conventional acupuncture; B, massage; C, analgesic; D, embedding needle therapy; E, acupressure; F, placebo; G, electroacupuncture; H, cupping; I, auricular acupuncture; J, acupoint implantation; K, acupoint injection; L, traditional Chinese medicine; and M, laser acupuncture.

The results of the NMA indicated that compared with placebo, conventional acupuncture (SMD: 1.22, 95% CI, 0.74 to 1.69) and electroacupuncture (SMD: 1.21, 95% CI, 0.14 to 2.26) could significantly reduce VAS scores, while conventional acupuncture plus cupping (SMD: −2.20, 95% CI, −3.54 to −0.94), auricular acupuncture (SMD: −1.40, 95% CI, −2.24 to −0.42), acupoint implantation (SMD: −2.48, 95% CI, −4.16 to −0.76), and acupoint injection (SMD: −3.75, 95% CI, −4.95 to −2.55) showed weaker effect in reducing VAS scores. The VAS scores of conventional acupuncture plus cupping (SMD: 1.50, 95% CI, 0.47 to 2.53) were significantly lower than that of analgesic, but the results showed that analgesic might gain a larger reduction in VAS scores than acupoint implantation (SMD: −1.78, 95% CI, −3.42 to −0.02) and acupoint injection (SMD: −3.04, 95% CI, −4.22 to −1.84). When compared to acupoint injection, conventional acupuncture (SMD: −2.53, 95% CI, −3.74 to −1.31), electroacupuncture (SMD: −2.55, 95% CI, −4.08 to −1.12), and auricular acupuncture (SMD: −2.34 95% CI, −3.88 to −0.91) had a better effect on decreasing VAS scores. In addition, four treatments including conventional acupuncture (SMD: 1.93, 95% CI, 0.03 to 3.84), conventional acupuncture plus cupping (SMD: 2.90, 95% CI, 0.67 to 5.22), acupoint implantation (SMD: 3.19, 95% CI, 0.63 to 5.71) and acupoint injection (SMD: 4.44, 95% CI, 2.23 to 6.74) showed superiority over traditional Chinese medicine, while conventional acupuncture plus traditional Chinese medicine (SMD: −2.70, 95% CI, −4.77 to −6.03) showed weaker effect in reducing VAS scores (Figure 5).

#### The number of migraine days

The results showed that conventional acupuncture (SMD: 2.03, 95% CI, 0.36 to 3.77) was superior to placebo, while electroacupuncture (SMD: −3.43, 95% CI, −6.76 to −0.05) had a lower effect than placebo (Figure 5).

#### Duration of migraine

From the network meta-analysis, we found that conventional acupuncture plus massage (SMD: −18.25, 95% CI, −28.23 to −7.56), analgesic (SMD: −21.99, 95% CI, −30.46 to −13.33), and conventional acupuncture (SMD: −20.36, 95% CI, −29.18 to −10.62) was superior to embedding needle therapy in their ability to reduce the duration of migraine. However, the results of the NMA indicated that compared with embedding needle therapy, acupressure (SMD: 17.91, 95% CI, 4.33 to 34.12), placebo (SMD: 22.94, 95% CI, 14.51 to 34.47), acupoint injection (SMD: 16.89, 95% CI, 7.70 to 27.70) and electroacupuncture (SMD: 21.30, 95% CI, 10.71 to 31.65) showed a weaker effect in reducing the duration of migraine. When compared to placebo, conventional acupuncture (SMD: 2.45, 95% CI, 0.48 to 9.45) had a better effect on decreasing the duration of migraine, while placebo was significantly more effective than acupoint injection (SMD: −6.00, 95% CI, −12.40 to −1.92) (Figure 5).

#### Frequency of migraine attacks

The results of NMA suggested that the comparative effectiveness of conventional acupuncture (SMD: 0.95, 95% CI, 0.14 to 1.81) was significantly better than placebo in the frequency of migraine attacks. The NMA showed that analgesic (SMD: −3.35, 95% CI, −5.29 to −1.46), conventional acupuncture (SMD: −3.05, 95% CI, −5.02 to −1.09), electroacupuncture (SMD: −4.00, 95% CI, −6.44 to −1.63), placebo (SMD: −4.01, 95% CI, −5.99 to −2.11), and acupressure plus analgesic (SMD: −3.23, 95% CI, −6.32 to −0.17) might gain a larger reduction in VAS scores than acupoint injection (Figure 5).

### Ranking

#### VAS scores

**Figure 6:**
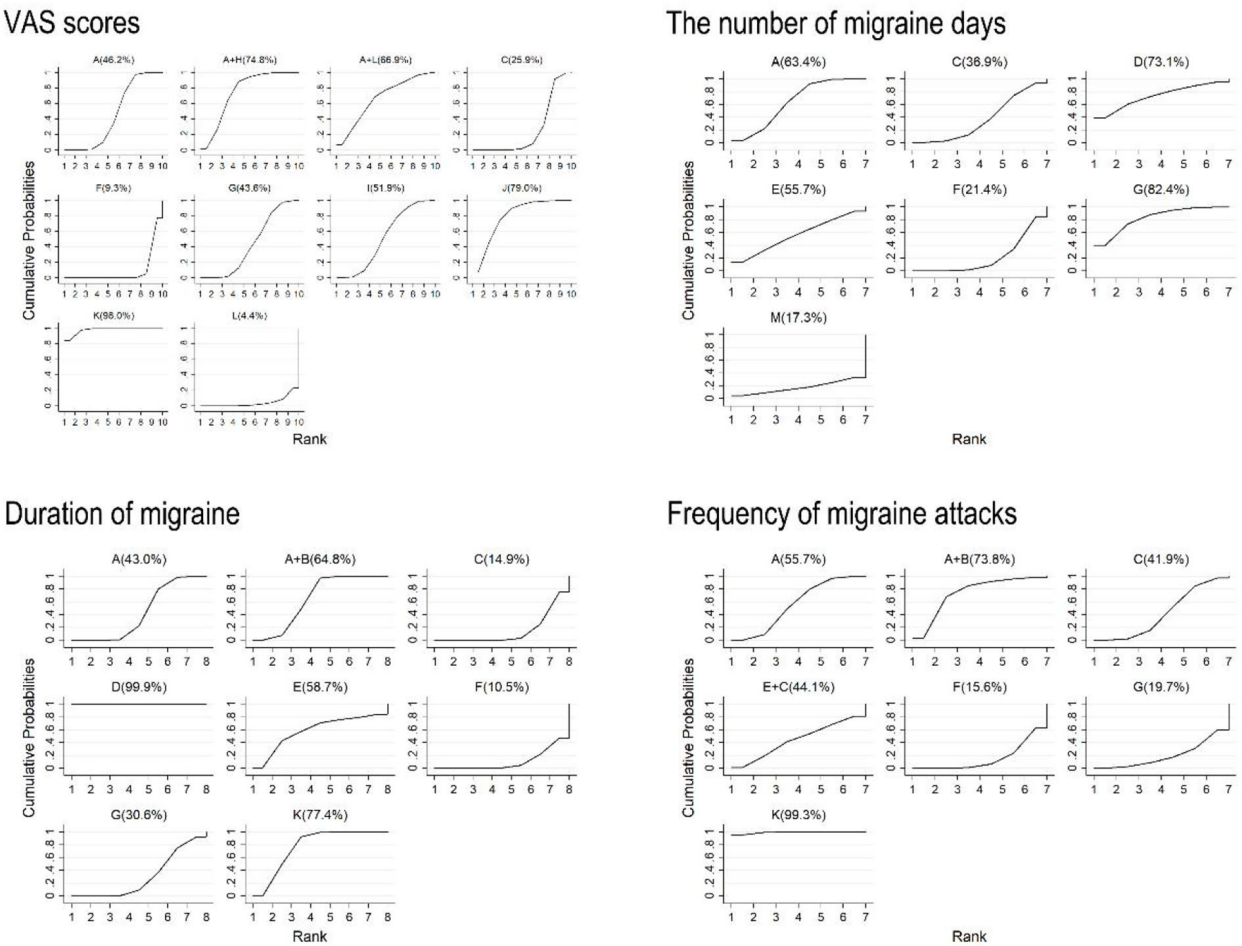
Surface under the cumulative ranking curves of VAS scores, the number of migraine days, duration of migraine, and frequency of migraine attacks. A, conventional acupuncture; B, massage; C, analgesic; D, embedding needle therapy; E, acupressure; F, placebo; G, electroacupuncture; H, cupping; I, auricular acupuncture; J, acupoint implantation; K, acupoint injection; L, traditional Chinese medicine; and M, laser acupuncture.

An estimated probability of ranking was measured by the SUCRA, which is displayed in Figure 6. With regard to the VAS scores, acupoint injection was ranked the highest (98.0%), followed by acupoint implantation (79.0%), conventional acupuncture plus cupping (74.8%), conventional acupuncture plus traditional Chinese medicine (66.9%), auricular acupuncture (51.9%), conventional acupuncture (46.2%), electroacupuncture (43.6%), analgesic (25.9%), and placebo (9.3%). Traditional Chinese medicine (4.4%) was ranked as the worst.

#### The number of migraine days

The results of the number of migraine days showed that electroacupuncture was the optimal intervention method (82.4%), followed by embedding needle therapy (73.1%), conventional acupuncture (63.4%), acupressure (55.7%), analgesic (36.9%), placebo (21.4%), and laser acupuncture (17.3%), which was ranked as the worst (Figure 6).

#### Duration of migraine

In terms of duration of migraine, the results indicated that embedding needle therapy ranked first (99.9%), the subsequent treatments were acupoint injection (77.4%), conventional acupuncture plus massage (64.8%), acupressure (58.7%), conventional acupuncture (43.0%), electroacupuncture (30.6%), analgesic (14.9%), and placebo (10.5%), which was ranked as the worst (Figure 6).

#### Frequency of migraine attacks

Ranking of the different types of acupuncture and related therapies is presented in Figure 6. As for the comparison in decreasing frequency of migraine attacks, acupoint injection was the best intervention (99.3%), followed by conventional acupuncture plus massage (73.8%), conventional acupuncture (55.7%), acupressure plus analgesic (44.1%), analgesic (41.9%), and electroacupuncture (19.7%). Placebo (15.6%) was ranked as the worst.

### Inconsistency assessment

#### VAS scores

From the network meta-analysis based on the inconsistency model, we found that there were three loops (i.e., Conventional acupuncture -Placebo -Auricular acupuncture, Conventional acupuncture -Analgesic -Placebo -Electroacupuncture, Conventional acupuncture -Analgesic -Placebo -Acupoint injection) of 95% CI including 0, which indicated that no obvious inconsistency was found in VAS scores. But the results also suggested that there was a statistical inconsistency between the direct comparison and the indirect comparison in one loop (i.e., Analgesic -Placebo -Electroacupuncture -Acupoint injection) (Supplementary Figure 1).

#### The number of migraine days

As shown in the figure, 95% CI of all loops (i.e., Conventional acupuncture -Placebo -Electroacupuncture, Conventional acupuncture -Acupressure -Placebo, Conventional acupuncture -Analgesic -Placebo) were included 0, which reflected that no obvious inconsistency was found in days with migraine (Supplementary Figure 1).

#### Duration of migraine

As for the inconsistency test outcome of the duration of migraine, there were two loops (i.e., Conventional acupuncture -Acupressure -Placebo, Conventional acupuncture -Conventional acupuncture plus massage -Analgesic) of 95% CI including 0, which suggested that no significant inconsistency was found. But the results showed that there was a statistical inconsistency between the direct comparison and the indirect comparison in one loop (i.e., Conventional acupuncture -Analgesic -Placebo -Acupoint injection) (Supplementary Figure 1).

#### Frequency of migraine attacks

The results indicated that there was one loop (i.e., Conventional acupuncture -Analgesic -Placebo -Acupoint injection) of 95% CI including 0, which illustrated that the evidence of inconsistency was not observed. However, three loops (i.e., Analgesic -Placebo -Electroacupuncture -Acupoint injection, Conventional acupuncture -Analgesic -Placebo -Electroacupuncture, Conventional acupuncture - Conventional acupuncture plus massage -Analgesic) were found to have significant inconsistency (Supplementary Figure 1).

### Adverse events

A total of 17 studies (34, 36, 39–43, 52, 54, 58–60, 62, 63, 66, 70, 71) out of 39 included RCTs reported adverse effect rates, all reported adverse events were of minor and transient nature. Fatigue and somnolence were observed in eight RCTs (34, 54, 58–60, 63, 70, 71). There were 9 studies (36, 40–43, 54, 63, 66, 70) reporting mild bleeding and hematoma. Moreover, four studies mentioned dizziness or nausea (39–41, 58), of which 2 studies (39, 41) reported pain in local skin after acupuncture treatment. There were thirteen included studies (35, 44, 46–49, 53, 55, 61, 64, 65, 68, 69) reporting no adverse events in both experimental group and placebo group. In conclusion, there were mild adverse reactions but no serious adverse reactions in the treatment of migraine by different acupuncture treatments. Detailed information is summarized in Table 1.

### Small sample size effect

The results showed that the funnel plots were not completely symmetrical, there may be a small sample effect, resulting in some publication bias in the included RCTs (Supplementary Figure 2).

## Discussion

The purpose of this network meta-analysis was to identify the efficacy and safety of acupuncture-related therapy for improving the symptoms of migraine. The present NMA provides evidence based on current clinical trials so that direct and indirect evidence from 39 RCTs in 4,379 migraine patients could be combined to compare the association of each acupuncture-related therapy with relative relief of migraine and adverse events. The results of the comparison demonstrated some essential findings. First of all, compared with a placebo, conventional acupuncture and electroacupuncture were significantly more effective in reducing VAS scores, while conventional acupuncture had a better effect on decreasing the number of migraine days, duration of migraine, and frequency of migraine attacks. Secondly, conventional acupuncture plus cupping was superior to analgesic to ameliorate VAS scores. Thirdly, based on the primary outcome in ranking graphs, acupoint injection was the most efficacious in improving VAS scores and frequency of migraine attacks. Probability ranking results in improving VAS scores showed that acupoint injection > acupoint implantation > conventional acupuncture plus cupping > conventional acupuncture plus traditional Chinese medicine > auricular acupuncture > conventional acupuncture > electroacupuncture > analgesic > placebo > traditional Chinese medicine, and probability ranking results in improving frequency of migraine attacks showed that acupoint injection > conventional acupuncture plus massage > conventional acupuncture > acupressure plus analgesic > analgesic > electroacupuncture > placebo. The results indicated that acupoint injection was superior to analgesic and placebo in improving VAS scores. As for the comparison in decreasing the number of migraine days, electroacupuncture ranked the optimal method, and embedding needle therapy was the most effective in lowering the duration of migraine. Probability ranking results in improving the number of migraine days showed that electroacupuncture > embedding needle therapy > conventional acupuncture > acupressure > analgesic > placebo > laser acupuncture, and probability ranking results in improving duration of migraine showed that embedding needle therapy > acupoint injection > conventional acupuncture plus massage > acupressure > conventional acupuncture > electroacupuncture > analgesic > placebo. The results also showed that electroacupuncture had a better effect on the number of migraine days than that of analgesic and placebo, and embedding needle therapy was significantly more effective in the duration of migraine than that of analgesic and placebo. The above results revealed that conventional acupuncture and acupoint injection may be useful clinical interventions to improve migraine.

Migraine is a recurrent disease, usually analgesic as the first choice to alleviate the pain of migraine attacks (72). Evidence of direct comparison and indirect comparison demonstrated that acupuncture and related therapies, acupuncture combined therapies (such as conventional acupuncture plus cupping) showed greater effectiveness than analgesic to relieve the pain of migraine patients. Results of pairwise meta-analyses also suggested that acupuncture and related therapies, acupuncture combined therapies (such as conventional acupuncture plus cupping, conventional acupuncture plus massage) were found to gain a larger reduction in the improvement of migraine symptoms when compared with analgesic. In addition to our research evidence, a large number of clinical studies and experimental studies have illustrated that acupuncture brings about a long-term effect in the treatment of migraine and has been recognized as an effective supplement or alternative therapy to relieve and prevent migraine attacks (41, 73). Experimental studies have reported that the neurotransmitter serotonin (5-HT) is involved in the onset of migraine, while acupuncture can promote the release of endogenous opioids by stimulating free nerve endings of pain receptors associated with pain control, and stimulate neurotransmitters such as 5-HT and β-endorphin in analgesic systems (74, 75). Additionally, it is a potential mechanism that electroacupuncture can relieve pain by inhibiting 5-HT7R in the descending pain modulatory system to improve central sensitization in migraine (76). Apart from the above experimental results, other previous studies have also pointed out that the effect of acupuncture to prevent migraine is related to the myosin light chain kinase in the middle meningeal artery that activates the migraine experimental model, and acupuncture can also reduce plasma glutamine levels in rats to relieve pain during acute migraine attacks (77, 78). Similarly, in response to local inflammation caused by pain, acupuncture can increase the body's immune function, resulting in a significant decrease in serum inflammatory factors such as TNF and IFN, so as to reduce pain and edema (79). Consistent with our existing results, most studies report that acupuncture-related therapy may have stronger effects and expectations than analgesics in clinical trials (33, 34, 37, 38, 42, 43, 45, 51, 58–60, 62, 67, 70, 71) and have a better effect than placebo (35, 36, 39–42, 44, 46–49, 52–55, 61, 63–66, 68, 69). Acupuncture as a preventive measure for migraine is safer and more effective than common medicines due to less side effects and lower risks and has been widely accepted in Western countries (33).

The NMA has several advantages. First, the results of the direct comparisons and indirect comparisons were taken into account in the network meta-analysis, which was beneficial to assess the treatments of migraine and provided a useful and complete description with the increasing sample size for the final pooled analysis. Second, Bayesian framework was conducted to compare acupuncture-related therapy with the main drug therapies for migraine treatment, and a formal rank order was provided for different acupuncture interventions by testing effectiveness *via* the SUCRA value. Last, seven different acupuncture therapies (conventional acupuncture, embedding needle therapy, acupressure, electroacupuncture, auricular acupuncture, acupoint implantation, and acupoint injection) were included to compare their effects on migraine treatment through a comprehensive search process and strict screening criteria. In order to reduce concerns about potential inconsistencies, we conducted a heterogeneity analysis to ensure the quality of this study.

Based on the results of the studies included in this paper, it can be seen that standard drug therapy (such as analgesics) and acupuncture are usually used as treatment measures for acute migraine. Regarding acupuncture, patients need to visit medical institutions frequently during the treatment process, which limits its universality in clinical practice and has certain limitations (58). Thus, for the treatment of acute migraine, doctors and most patients prefer to choose analgesics which can improve symptoms quickly to achieve a faster and more thorough treatment effect (80). Although commonly used medications such as triptans, ergotamine, and barbiturates have positive therapeutic effects on migraine, the side effects of drug therapy such as weight gain, fatigue, sleep disturbance, and gastrointestinal intolerance are common in patients with long-term use (81). The included studies showed that acupuncture was favored for migraine prevention in terms of efficacy. With regards to drug therapy, acupuncture was superior to medication in reducing headache frequency and pain severity, migraine symptoms, and adverse events. For migraine patients who need preventive treatment due to frequent migraine attacks or poor control, especially those who refuse preventive drug treatment or suffer from adverse events, as well as those who are intolerant of drugs, acupuncture should be considered as a treatment option (82). Therefore, it is necessary to classify different types of migraine treatment measures in detail to provide a more refined and targeted treatment plan for clinical practice. In addition, the types of drug therapy for migraine used in this study varied, including analgesics (e.g. ergotamine, ibuprofen, gabapentin) and preventive drug (e.g. flunarizine hydrochloride, valproic acid, topiramate, metoprolol). Although the guidelines mainly recommend drug treatment such as acute specific medications (e.g. Triptans, Ditans, Gepants) and preventive specific medications (e.g. Gepants, Anti-CGRP mAbs), there are still some drugs that are not specifically used to treat migraine (83). The drug dosage was inconsistent in each study. Because the condition of most migraine patients is constantly changing, it is difficult to ensure that the drug type and dosage of each patient are consistent throughout the treatment period. In addition, physicians have different treatment habits. Some physicians prefer to use combination drugs to treat migraine to quickly relieve pain, which also increases the difficulty of studying and comparing the same drug treatment methods (80). In the treatment process, the comparison between acupuncture and drug treatment is difficult to be blinded, and there is a risk of bias (84). Therefore, it is inappropriate to compare acupuncture for preventive migraine treatment with analgesics for acute migraine treatment. Future research should focus on the frequency, intensity, and duration of acupuncture treatment. As well as further standardization of the selection of drug treatment types, time and dosage, this is also the focus of further improvement and optimization of the experimental design in the randomized controlled clinical trials of acupuncture in the treatment of migraine in the future.

The results of this paper show that acupuncture-related therapy is more effective in improving migraine than the placebo group. In addition to acupuncture, the placebo group included in the study also had sham acupuncture (acupuncture needles are movable devices, the tips of which can be retracted into the handle as the needles are inserted; acupuncture does not actually penetrate the surface of the skin), acupuncture non-therapeutic acupoints (points that have no therapeutic effect on migraine are selected, or acupuncture is performed on areas of the skin that do not have acupoints), sham acupuncture without stimulation, or shallow acupuncture (inserting acupuncture needles into the subcutaneous layer shallowly without manual stimulation), injecting normal saline into sham acupoints, etc. This also shows that there is no uniform standard for the placebo control group of acupuncture in clinical studies. It is doubtful whether they penetrate the skin, or whether they are far away from the real acupoints or the area around the acupoints. In addition, acupuncture-related physiological effects may be produced whether inserted into acupoints or non-acupoints (66). In relevant trials where real acupuncture and sham acupuncture had similar effects, it could not be determined that acupuncture had only a placebo effect. A previous study also showed that placebo needles can stimulate unmyelinated afferent nerves, even if the acupuncture needle is not inserted, it will also affect pain transmission (85). Therefore, needle insertion into superficial skin areas cannot simply be considered a normative placebo control (86). In addition, the quality of evidence in the included studies ranges from low to medium, so it is not entirely certain whether the included patients had never received acupuncture, which would affect the blinded evaluation of the intervention. However, some placebo sham acupuncture without stimulation will still pierce the skin, which may also affect the success of blinding and cause bias. Piercing the skin also affects the success rate of blinding. This also fails to make the blinding psychologically and physiologically credible for the patient, and it is difficult to ensure complete blinding between the patient and the therapist, which makes the blinding ineffective. Therefore, in the placebo (sham acupuncture) design of future studies on acupuncture, more consideration should be given to the optimal design of blinding. For example, recruit patients who have not received acupuncture treatment, use non-puncture needles as placebo (sham acupuncture method) control, or design needles that meet the requirements of placebo to reduce blinding failure and efficacy bias caused by the above factors, so as to obtain high-quality research results.

In addition, this article provides a slight reference to the comparison of acute-onset and chronic migraine, but some of the included studies do not mention specific migraine types. Medications are often the first-line treatment for migraine, but they also have many side effects, which will aggravate the headache of chronic migraine patients (87). Patients who regularly take acute medications for headache attacks may develop chronic headaches and medication-overuse headaches later in life. At present, there is no clear explanation for the mechanism of chronic migraine, but studies have shown that calcitonin gene–related peptide (CGRP) in the circulation of patients is persistently elevated during chronic migraine (88). In addition, central sensitization is thought to be one of the key mechanisms of migraine chronicity (89). Mechanism studies suggested that acupuncture may exert anti-inflammatory effects by releasing neuropeptides, including CGRP, from nerve endings, thereby improving and preventing the occurrence and symptoms of migraine. Another study shows that acupuncture may play an analgesic role through the hypothalamic–pituitary–adrenal axis and the endogenous opioid system, which is an important mediator of pain stress response (90). This study also shows that acupuncture, as a preventive treatment for chronic migraine, can reduce the number of days and duration of headache, and gradually reduce the frequency of using analgesic. Acupuncture can also be considered a treatment option for patients with drug overuse. Some studies have shown that for migraine patients who do not respond to acute drugs, acupuncture can achieve its analgesic effect by regulating Aσ-fibers, which may become an important complementary and alternative therapy for migraine (91). Unfortunately, only 5 studies of chronic migraine and 4 studies of acute-onset migraine are mentioned in this article, and no studies involving migraine with drug overuse. Therefore, there is insufficient evidence to provide further support for the clinical value of acupuncture. In future work, further and more in-depth research should be carried out on the specific pathophysiological mechanism of drug overuse of migraine and acupuncture treatment of acute and chronic migraine.

Nevertheless, there are some limitations in the present study. Firstly, as a result of lacking statistics of large numbers of studies, the study only provided partial data on the frequency of common adverse events, which makes it impossible to accurately assess the safety of different types of acupoint stimulation therapy, and the research quality would be usually affected to some extent. Secondly, we only evaluated the overall efficacy and safety of acupuncture-related therapy, and the acupoint selection between acupuncture treatments may differ from each other or be empirical. Future clinical studies may focus on the different effects of specific acupoints on migraine treatment, which deserves further exploration. Thirdly, the sample size of some studies was too small, resulting in lower levels of evidence. Fourth, the sessions of acupuncture are not uniform, and the follow-up time ranges from 4 to 12 weeks. Since migraine is a recurrent headache, it is necessary to conduct longer follow-up to observe the long-term effects of acupuncture on migraine. Finally, the majority of the included studies lacked the use of blinding, which may lead to publication bias.

## Conclusion

In conclusion, the existing evidence demonstrates that conventional acupuncture and electroacupuncture show superiority in reducing VAS scores, and conventional acupuncture shows superiority in decreasing the number of migraine days, duration of migraine, and frequency of migraine attacks when compared with the placebo. Conventional acupuncture plus cupping is superior to analgesic in their ability to ameliorate VAS scores. Overall, acupuncture and related therapies could be recommended as one of the effective treatments for migraine, conventional acupuncture has a significant effect in improving four efficacy outcomes of migraine, and acupoint injection rank as the optimal method for both improving VAS scores and frequency of migraine attacks. In addition to the mentioned above, electroacupuncture is the most effective in reducing the number of migraine days and embedding needle therapy ranks first in decreasing the duration of migraine. Conventional acupuncture, acupoint injection, electroacupuncture, and embedding needle therapy are significantly more effective than that of analgesic and placebo improving migraine. In clinical practice, appropriate treatments should be selected based on the condition of patient. Further high-quality, large-sample, multicenter randomized controlled trials are needed to validate these findings.

## Data availability statement

The original contributions presented in the study are included in the article/Supplementary material, further inquiries can be directed to the corresponding author.

## Author contributions

YY and FL: study design. YS and TL: data collection and writing-original draft. YS and HL: full-text review and data extraction. CM and TL: investigation. YS, TL, and HL: statistical analyses. YS, TL, CM, FL, and YY: contribution to and review of the final version of the manuscript. All authors contributed to the article and approved the submitted version.

## Funding

This work was supported by the National Natural Science Foundation of China (Nos. 81970261, 82100440); Natural Science Foundation of Hubei Province (No. 2021CFB496); Research and innovation team project of Wuhan Sports University (No. 21KT04); Advantageous and Characteristic Disciplines (Groups) of Colleges and Universities in Hubei Province during the 14th Five Year Plan.

## Conflict of interest

The authors declare that the research was conducted in the absence of any commercial or financial relationships that could be construed as a potential conflict of interest.

## Publisher's note

All claims expressed in this article are solely those of the authors and do not necessarily represent those of their affiliated organizations, or those of the publisher, the editors and the reviewers. Any product that may be evaluated in this article, or claim that may be made by its manufacturer, is not guaranteed or endorsed by the publisher.
