# Supplementary material for: Comparative efficacy of acupuncture-related therapy for migraine: A systematic review and network meta-analysis
Source: Front Neurol. 2022 Oct 26;13:1010410. doi: 10.3389/fneur.2022.1010410 (PMC9643721; doi:10.3389/fneur.2022.1010410)
Supplement: Supplementary file 1 [file Data_Sheet_1.pdf]

Supplementary Material

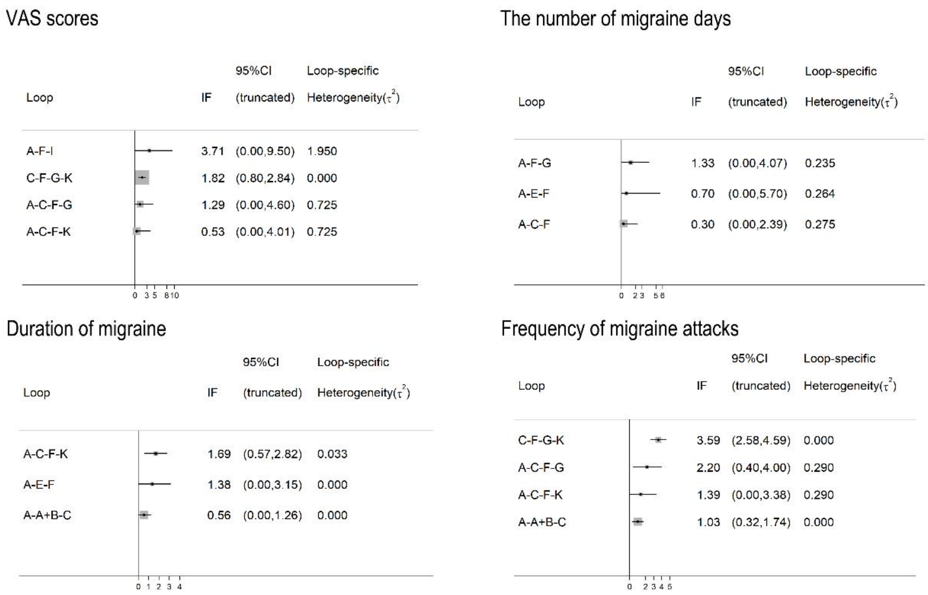

Supplementary Figure 1. Inconsistency assessment
(If 95% CI of loops were included 0, which reflected that no significant inconsistency was found.)

Notes: A, conventional acupuncture; B, massage; C, analgesic; D, embedding needle therapy; E, acupressure; F, placebo; G, electroacupuncture; H, cupping; I, auricular acupuncture; J, acupoint implantation; K, acupoint injection; L, traditional chinese medicine; M, laser acupuncture.

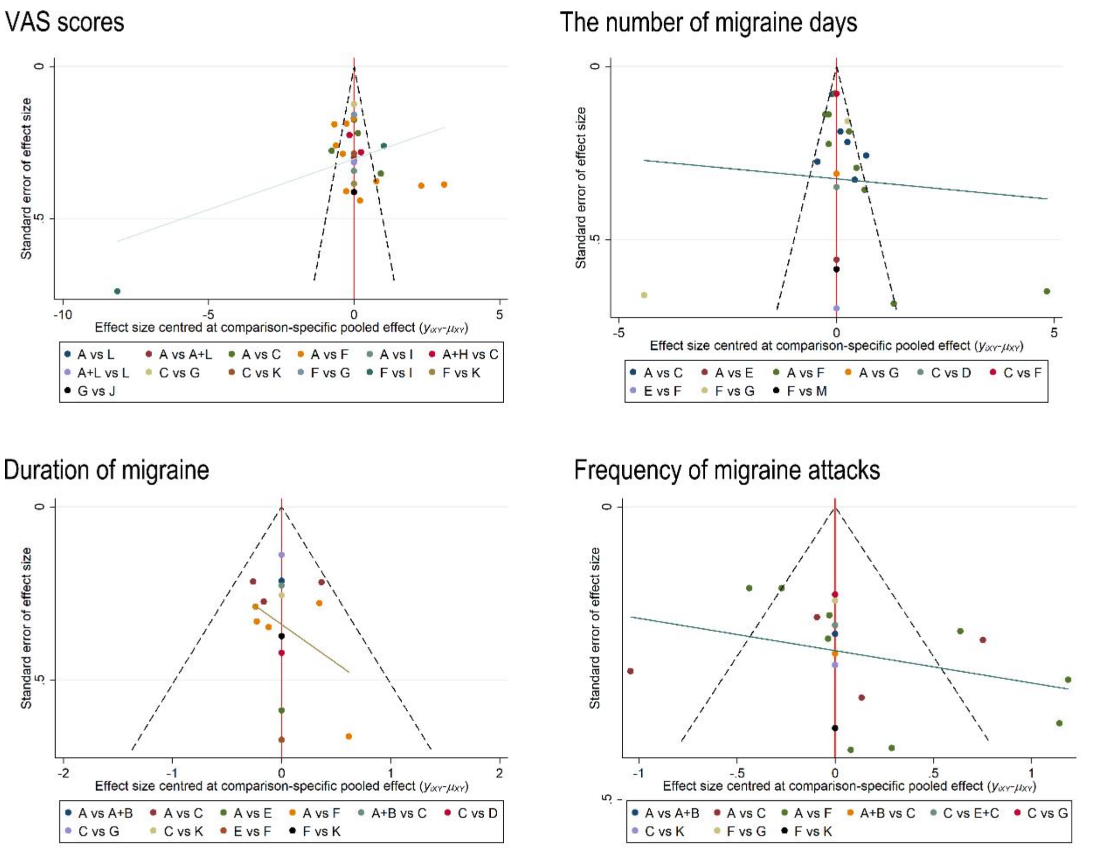

Supplementary Figure 2. The funnel plots of each outcome for the network meta-analysis.

Notes: A, conventional acupuncture; B, massage; C, analgesic; D, embedding needle therapy; E, acupressure; F, placebo; G, electroacupuncture; H, cupping; I, auricular acupuncture; J, acupoint implantation; K, acupoint injection; L, traditional chinese medicine; M, laser acupuncture.

Additional file 1: the PRISMA-NMA checklist treatment groups for included studies in a network meta-analysis

Section/Topic	Item #	Checklist Item	Reported on Page #
**TITLE**			
Title	1	Identify the report as a systematic review *incorporating a network meta-analysis (or related form of meta-analysis).*	1
**ABSTRACT**			
Structured summary	2	Provide a structured summary including, as applicable:<br>**Background:** main objectives<br>**Methods:** data sources; study eligibility criteria, participants, and interventions; study appraisal; and *synthesis methods, such as network meta-analysis.*<br>**Results:** number of studies and participants identified; summary estimates with corresponding confidence/credible intervals; *treatment rankings may also be discussed. Authors may choose to summarize pairwise comparisons against a chosen treatment included in their analyses for brevity.*<br>**Discussion/Conclusions:** limitations; conclusions and implications of findings.<br>**Other:** primary source of funding; systematic review registration number with registry name.	2-3
**INTRODUCTION**			
Rationale	3	Describe the rationale for the review in the context of what is already known*, including mention of why a network meta-analysis has been conducted.*_	4
Objectives	4	Provide an explicit statement of questions being addressed, with reference to participants, interventions, comparisons, outcomes, and study design (PICOS).	4-5
**METHODS**			
Protocol and registration	5	Indicate whether a review protocol exists and if and where it can be accessed (e.g., Web address); and, if available, provide registration information, including registration number.	6
Eligibility criteria	6	Specify study characteristics (e.g., PICOS, length of follow-up) and report characteristics (e.g., years considered, language, publication status) used as criteria for eligibility, giving rationale. *Clearly describe eligible treatments included in the treatment network, and note whether any have been clustered or merged into the same node (with justification).*_	6

Information sources	7	Describe all information sources (e.g., databases with dates of coverage, contact with study authors to identify additional studies) in the search and date last searched.	6
Search	8	Present full electronic search strategy for at least one database, including any limits used, such that it could be repeated.	6
Study selection	9	State the process for selecting studies (i.e., screening, eligibility, included in systematic review, and, if applicable, included in the meta-analysis).	6
Data collection process	10	Describe method of data extraction from reports (e.g., piloted forms, independently, in duplicate) and any processes for obtaining and confirming data from investigators.	6-7
Data items	11	List and define all variables for which data were sought (e.g., PICOS, funding sources) and any assumptions and simplifications made.	7
**Geometry of the network**	**S1**	Describe methods used to explore the geometry of the treatment network under study and potential biases related to it. This should include how the evidence base has been graphically summarized for presentation, and what characteristics were compiled and used to describe the evidence base to readers.	7-8
Risk of bias within individual studies	12	Describe methods used for assessing risk of bias of individual studies (including specification of whether this was done at the study or outcome level), and how this information is to be used in any data synthesis.	8-9
Summary measures	13	State the principal summary measures (e.g., risk ratio, difference in means). *Also describe the use of additional summary measures assessed, such as treatment rankings and surface under the cumulative ranking curve (SUCRA) values, as well as modified approaches used to present summary findings from meta-analyses.*	8-9
Planned methods of analysis	14	Describe the methods of handling data and combining results of studies for each network meta-analysis. This should include, but not be limited to:<br>• *Handling of multi-arm trials;*<br>• *Selection of variance structure;*<br>• *Selection of prior distributions in Bayesian analyses; and*<br>• *Assessment of model fit.*	8-9
**Assessment of Inconsistency**	**S2**	Describe the statistical methods used to evaluate the agreement of direct and indirect evidence in the treatment network(s) studied. Describe efforts taken to address its presence when found.	8
Risk of bias across studies	15	Specify any assessment of risk of bias that may affect the cumulative evidence (e.g., publication bias, selective reporting within studies).	8-9

Additional analyses	16	Describe methods of additional analyses if done, indicating which were pre-specified. This may include, but not be limited to, the following:<br>• Sensitivity or subgroup analyses;<br>• Meta-regression analyses;<br>• *Alternative formulations of the treatment network; and*<br>• *Use of alternative prior distributions for Bayesian analyses (if applicable).*_	8-9
**RESULTS†**			
Study selection	17	Give numbers of studies screened, assessed for eligibility, and included in the review, with reasons for exclusions at each stage, ideally with a flow diagram.	10
**Presentation of network structure**	**S3**	Provide a network graph of the included studies to enable visualization of the geometry of the treatment network.	10，Figure 4
**Summary of network geometry**	**S4**	Provide a brief overview of characteristics of the treatment network. This may include commentary on the abundance of trials and randomized patients for the different interventions and pairwise comparisons in the network, gaps of evidence in the treatment network, and potential biases reflected by the network structure.	10, Figure 4
Study characteristics	18	For each study, present characteristics for which data were extracted (e.g., study size, PICOS, follow-up period) and provide the citations.	10-11, Table 1，Table 2
Risk of bias within studies	19	Present data on risk of bias of each study and, if available, any outcome level assessment.	11, Figure 2, Figure 3
Results of individual studies	20	For all outcomes considered (benefits or harms), present, for each study: 1) simple summary data for each intervention group, and 2) effect estimates and confidence intervals. *Modified approaches may be needed to deal with information from larger networks.*	10-11, Table3
Synthesis of results	21	Present results of each meta-analysis done, including confidence/credible intervals. *In larger networks, authors may focus on comparisons versus a particular comparator (e.g. placebo or standard care), with full findings presented in an appendix. League tables and forest plots may be considered to summarize pairwise comparisons.* If additional summary measures were explored (such as treatment	11-17, Table 4, Figure 5, Figure 6

		rankings), these should also be presented.	
**Exploration for inconsistency**	**S5**	Describe results from investigations of inconsistency. This may include such information as measures of model fit to compare consistency and inconsistency models, *P* values from statistical tests, or summary of inconsistency estimates from different parts of the treatment network.	16-17
Risk of bias across studies	22	Present results of any assessment of risk of bias across studies for the evidence base being studied.	11, Figure 2, Figure 3
Results of additional analyses	23	Give results of additional analyses, if done (e.g., sensitivity or subgroup analyses, meta-regression analyses*, alternative network geometries studied, alternative choice of prior distributions for Bayesian analyses,* and so forth).	17-18
**DISCUSSION**			
Summary of evidence	24	Summarize the main findings, including the strength of evidence for each main outcome; consider their relevance to key groups (e.g., healthcare providers, users, and policy-makers).	19-20
Limitations	25	Discuss limitations at study and outcome level (e.g., risk of bias), and at review level (e.g., incomplete retrieval of identified research, reporting bias). Comment on the validity of the assumptions, such as transitivity and consistency. Comment on any concerns regarding network geometry (e.g., avoidance of certain comparisons).	25
Conclusions	26	Provide a general interpretation of the results in the context of other evidence, and implications for future research.	27
**FUNDING**			
Funding	27	Describe sources of funding for the systematic review and other support (e.g., supply of data); role of funders for the systematic review. This should also include information regarding whether funding has been received from manufacturers of treatments in the network and/or whether some of the authors are content experts with professional conflicts of interest that could affect use of treatments in the network.	28

Abbreviations: *N/A* not applicable, *S* specific to reporting of network meta-analyses that has been added to guidance from the PRISMA statement, *PICOS* population intervention comparators outcomes study design.

Additional file 2: Search strategies for RCTs on acupuncture for migraine

PubMed:

#1 "Migraine Disorders"[Mesh]

#2 Disorder, Migraine[Title/Abstract] OR Disorders, Migraine [Title/Abstract] OR Migraine Disorder [Title/Abstract] OR Migraine [Title/Abstract] OR Migraines[Title/Abstract] OR Migraine Headache [Title/Abstract] OR Headache, Migraine[Title/Abstract] OR Headaches, Migraine [Title/Abstract] OR Migraine Headaches[Title/Abstract] OR Acute Confusional Migraine [Title/Abstract] OR Acute Confusional Migraines [Title/Abstract] OR Migraine, Acute Confusional [Title/Abstract] OR Migraines, Acute Confusional [Title/Abstract] OR Status Migrainosus [Title/Abstract] OR Hemicrania Migraine [Title/Abstract] OR Hemicrania Migraines [Title/Abstract] OR Migraine, Hemicrania [Title/Abstract] OR Migraines, Hemicrania [Title/Abstract] OR Migraine Variant [Title/Abstract] OR Migraine Variants [Title/Abstract] OR Variant, Migraine [Title/Abstract] OR Variants, Migraine [Title/Abstract] OR Sick Headache [Title/Abstract] OR Headache, Sick [Title/Abstract] OR Headaches, Sick [Title/Abstract] OR Sick Headaches [Title/Abstract] OR Abdominal Migraine [Title/Abstract] OR Abdominal Migraines [Title/Abstract] OR Migraine, Abdominal [Title/Abstract] OR Migraines, Abdominal [Title/Abstract] OR Cervical Migraine Syndrome [Title/Abstract] OR Cervical Migraine Syndromes [Title/Abstract] OR Migraine Syndrome, Cervical [Title/Abstract] OR Migraine Syndromes, Cervical [Title/Abstract]

#3 #1 OR #2

#4 "Acupuncture"[Mesh]

#5 "Acupuncture Therapy"[Mesh]

#6 "Acupuncture, Ear"[Mesh]

#7 "Acupuncture Points"[Mesh]

#8 "Acupuncture Analgesia"[Mesh]

#9 "Auriculotherapy" [Mesh]

#10 Acupuncture [Title/Abstract] OR moxibustion [Title/Abstract] OR acustimulation [Title/Abstract] OR Acupuncture Analgesia [Title/Abstract] OR silver needle [Title/Abstract] OR trigger point [Title/Abstract] OR de qi [Title/Abstract] OR electro-acupuncture [Title/Abstract] OR meridian [Title/Abstract] OR Auriculotherapy [Title/Abstract] OR Extra points [Title/Abstract] OR needle pricking [Title/Abstract] OR Transcutaneous Electric Nerve Stimulation [Title/Abstract] OR acupressure [Title/Abstract] OR needling [Title/Abstract] OR intradermal needle [Title/Abstract] OR Point application [Title/Abstract] OR fire needle [Title/Abstract] OR three-edged needle [Title/Abstract] OR a-shi point [Title/Abstract] OR five phase points [Title/Abstract] OR needle-embedding [Title/Abstract] OR pricking therapy [Title/Abstract] OR point injection [Title/Abstract] OR incision therapy [Title/Abstract] OR body-acupuncture [Title/Abstract] OR puncturing collateral [Title/Abstract] OR quick puncture [Title/Abstract]

#11 #4 OR #5 OR #6 OR #7 OR #8 OR #9 OR #10

#12 "Randomized Controlled Trial" [Publication Type]

#13 "Randomized Controlled Trials as Topic"[Mesh]

#14 "Pragmatic Clinical Trial" [Publication Type]

#15 "Pragmatic Clinical Trials as Topic"[Mesh]

#16 "Intention to Treat Analysis"[Mesh]

#17 "random allocation"[Mesh Terms]

#18 random*[Title/Abstract]

#19 #12 OR #13 OR #14 OR #15 OR #16 OR #17 OR #18

#20 #3 AND #11 AND #19

EMBASE:
#1 'migraine'/exp
#2 'Migraine Disorders':ti, ab OR 'Disorders,Migraine':ti,ab OR 'Migraine Disorder':ti,ab OR 'Migraine':ti,ab OR 'Migraines':ti,ab OR 'Migraine Headache':ti,ab OR 'Headache,Migraine':ti,ab OR 'Headaches,Migraine':ti,ab OR 'Migraine Headaches':ti,ab OR 'Acute Confusional Migraine':ti,ab OR 'Acute Confusional Migraines':ti,ab OR 'Migraine,Acute Confusional':ti,ab OR 'Migraines,Acute Confusional':ti,ab OR 'Status Migrainosus':ti,ab OR 'Hemicrania Migraine':ti,ab OR 'Hemicrania Migraines':ti,ab OR 'Migraine,Hemicrania':ti,ab OR 'Migraines, Hemicrania':ti,ab OR 'Migraine Variant':ti,ab OR 'Migraine Variants':ti,ab OR 'Variant,Migraine':ti,ab OR 'Variants,Migraine':ti,ab OR 'Sick Headache':ti,ab OR 'Headache,Sick':ti,ab OR 'Headaches,Sick':ti,ab OR 'Sick Headaches':ti,ab OR 'Abdominal Migraine':ti,ab OR 'Abdominal Migraines':ti,ab OR 'Migraine, Abdominal':ti,ab OR 'Migraines,Abdominal':ti,ab OR 'Cervical Migraine Syndrome':ti,ab OR 'Cervical Migraine Syndromes':ti,ab OR 'Migraine Syndrome, Cervical':ti,ab OR 'Migraine Syndromes,Cervical':ti,ab
#3 #1 OR #2
#4 'acupuncture'/exp
#5 'acupuncture needle'/exp
#6 'electroacupuncture '/exp
#7 'acupuncture':ti,ab OR 'moxibustion':ti,ab OR 'acustimulation':ti,ab OR 'acupuncture analgesia':ti,ab OR 'silver needle':ti,ab OR 'trigger point':ti,ab OR 'de qi':ti,ab OR 'electro-acupuncture':ti,ab OR 'meridian':ti,ab OR 'Auriculotherapy':ti,ab OR 'extra points':ti,ab OR 'needle pricking':ti,ab OR 'transcutaneous electric nerve stimulation':ti,ab OR 'acupressure':ti,ab OR 'needling':ti,ab OR 'intradermal needle':ti,ab OR 'point application':ti,ab OR 'fire needle':ti,ab OR 'three-edged needle':ti,ab OR 'a-shi point':ti,ab OR 'five phase points':ti,ab OR 'needle-embedding':ti,ab OR 'pricking therapy':ti,ab OR 'point injection':ti,ab OR 'incision therapy':ti,ab OR 'body-acupuncture':ti,ab OR 'puncturing collateral':ti,ab OR 'quick puncture':ti,ab
#8 #4 OR #5 OR #6 OR #7
#9 'randomized controlled trial'/exp
#10 'randomized controlled trial (topic)'/exp
#11 random*:ti,ab
#12 #9 OR #10 OR #11
#13 #3 AND #8 AND #12

Cochrane Library:
#1 MeSH descriptor: [Migraine Disorders] explode all trees
#2 'Migraine Disorders':ti,ab OR 'Disorders,Migraine':ti,ab OR 'Migraine Disorder':ti,ab OR 'Migraine':ti,ab OR 'Migraines':ti,ab OR 'Migraine Headache':ti,ab OR 'Headache,Migraine':ti,ab OR 'Headaches,Migraine':ti,ab OR 'Migraine Headaches':ti,ab OR 'Acute Confusional Migraine':ti,ab OR 'Acute Confusional Migraines':ti,ab OR 'Migraine,Acute Confusional':ti,ab OR 'Migraines,Acute Confusional':ti,ab OR 'Status Migrainosus':ti,ab OR 'Hemicrania Migraine':ti,ab OR 'Hemicrania Migraines':ti,ab OR 'Migraine,Hemicrania':ti,ab OR 'Migraines, Hemicrania':ti,ab OR 'Migraine Variant':ti,ab OR 'Migraine Variants':ti,ab OR 'Variant,Migraine':ti,ab OR 'Variants,Migraine':ti,ab OR 'Sick Headache':ti,ab OR

'Headache,Sick':ti,ab OR 'Headaches,Sick':ti,ab OR 'Sick Headaches':ti,ab OR 'Abdominal Migraine':ti,ab OR 'Abdominal Migraines':ti,ab OR 'Migraine, Abdominal':ti,ab OR 'Migraines,Abdominal':ti,ab OR 'Cervical Migraine Syndrome':ti,ab OR 'Cervical Migraine Syndromes':ti,ab OR 'Migraine Syndrome, Cervical':ti,ab OR 'Migraine Syndromes,Cervical':ti,ab
#3 #1 OR #2
#4 MeSH descriptor: [Acupuncture] explode all trees
#5 MeSH descriptor: [Acupuncture Therapy] explode all trees
#6 MeSH descriptor: [Acupuncture, Ear] explode all trees
#7 'Acupuncture':ti,ab OR 'moxibustion':ti,ab OR 'acustimulation':ti,ab OR 'Acupuncture Analgesia':ti,ab OR 'silver needle':ti,ab OR 'trigger point':ti,ab OR 'de qi':ti,ab OR 'electro-acupuncture':ti,ab OR 'meridian':ti,ab OR 'Auriculotherapy':ti,ab OR 'Extra points':ti,ab OR 'needle pricking':ti,ab OR 'Transcutaneous Electric Nerve Stimulation':ti,ab OR 'acupressure':ti,ab OR 'needling':ti,ab OR 'intradermal needle':ti,ab OR 'Point application':ti,ab OR 'fire needle':ti,ab OR 'three-edged needle':ti,ab OR 'a-shi point':ti,ab OR 'five phase points':ti,ab OR 'needle-embedding':ti,ab OR 'pricking therapy':ti,ab OR 'point injection':ti,ab OR 'incision therapy':ti,ab OR 'body-acupuncture':ti,ab OR 'puncturing collateral':ti,ab OR 'quick puncture':ti,ab
#8 #4 OR #5 OR #6 OR #7
#9 MeSH descriptor: [Randomized Controlled Trial] explode all trees
#10 MeSH descriptor: [Randomized Controlled Trials as Topic] explode all trees
#11 #9 OR #10
#12 #3 AND #8 AND #11

Web of Science:
#1 TS=(migraine disorders OR Disorder, Migraine OR Disorders, Migraine OR Migraine Disorder OR Migraine OR Migraines OR Migraine Headache OR Headache,Migraine OR Headaches,Migraine OR Migraine Headaches OR Acute Confusional Migraine OR Acute Confusional Migraines OR Migraine,Acute Confusional OR Migraines,Acute Confusional OR Status Migrainosus OR Hemicrania Migraine OR Hemicrania Migraines OR Migraine, Hemicrania OR Migraines,Hemicrania OR Migraine Variant OR Migraine Variants OR Variant, Migraine OR Variants,Migraine OR Sick Headache OR Headache,Sick OR Headaches,Sick OR Sick Headaches OR Abdominal Migraine OR Abdominal Migraines OR Migraine,Abdominal OR Migraines,Abdominal OR Cervical Migraine Syndrome OR Cervical Migraine Syndromes OR Migraine Syndrome, Cervical OR Migraine Syndromes,Cervical)
#2 TS=(Acupuncture OR Acupuncture Therapy OR Acupuncture,Ear OR Acupuncture Points OR Acupuncture Analgesia OR Auriculotherapy OR moxibustion OR acustimulation OR Acupuncture Analgesia OR silver needle OR trigger point OR de qi OR electro-acupuncture OR meridian OR Auriculotherapy OR Extra points OR needle pricking OR Transcutaneous Electric Nerve Stimulation OR acupressure OR needling OR intradermal needle OR Point application OR fire needle OR three-edged needle OR a-shi point OR five phase points OR needle-embedding OR pricking therapy OR point injection OR incision therapy OR body-acupuncture OR puncturing collateral OR quick puncture)
#3 TS=(Randomized Controlled Trial OR Randomized Controlled Trials as Topic OR Random)
#4 #1 AND #2 AND #3

CNKI
#1 (主题 = 偏头痛 或者 题名 = 偏头痛 或者 v_subject= 中英文扩展 (偏头痛) 或者 title= 中英文扩展 (偏头痛)) (模糊匹配)
#2 (主题 = 原发性头痛 或者 题名 = 原发性头痛 或者 v_subject= 中英文扩展 (原发性头痛) 或者 title= 中英文扩展 (原发性头痛)) (模糊匹配)
#3 (主题 = 头痛 或者 题名 = 头痛 或者 v_subject= 中英文扩展 (头痛) 或者 title= 中英文扩展 (头痛)) (模糊匹配)
#4 #1 OR #2 OR #3
#5 (主题 = 针刺 或者 题名 = 针刺 或者 v_subject= 中英文扩展 (针刺) 或者 title= 中英文扩展 (针刺)) (模糊匹配)
#6 (主题 = 针灸 或者 题名 = 针灸 或者 v_subject= 中英文扩展 (针灸) 或者 title= 中英文扩展 (针灸)) (模糊匹配)
#7 (主题 = 电针 或者 题名 = 电针 或者 v_subject= 中英文扩展 (电针) 或者 title= 中英文扩展 (电针)) (模糊匹配)
#8 (主题 = 头针 或者 题名 = 头针 或者 v_subject= 中英文扩展 (头针) 或者 title= 中英文扩展 (头针)) (模糊匹配)
#9 (主题 = 腕踝针 或者 题名 = 腕踝针 或者 v_subject= 中英文扩展 (腕踝针) 或者 title= 中英文扩展 (腕踝针)) (模糊匹配)
#10 (主题 = 平衡针 或者 题名 = 平衡针 或者 v_subject= 中英文扩展 (平衡针) 或者 title= 中英文扩展 (平衡针)) (模糊匹配)
#11 (主题 = 针 或者 题名 = 针 或者 v_subject= 中英文扩展 (针) 或者 title= 中英文扩展 (针)) (模糊匹配)
#12 #4 OR #5 OR #6 OR #7 OR #8 OR #9 OR #10 OR #11
#13 (摘要 = 随机 或者 abstract_en= 中英文扩展 (随机)) (模糊匹配)
#14 (摘要 =RCT 或者 abstract_en= 中英文扩展 (RCT)) (模糊匹配)
#15 #13 OR #14
#16 #4 AND #12 AND #15

Wanfang Database
主题 :(偏头痛 or 原发性头痛 or 头痛) and 主题 :(针刺 or 针灸 or 电针 or 头针 or 腕踝针 or 平衡针 or 针) and 主题 :(随机 or RCT)

VIP Database
((((题名或关键词 = 偏头痛 OR 题名或关键词 = 原发性头痛) OR 题名或关键词 = 头痛) AND ((((((题名或关键词 = 针刺 OR 题名或关键词 = 针灸) OR 题名或关键词 = 电针) OR 题名或关键词 = 头针) OR 题名或关键词 = 腕踝针) OR 题名或关键词 = 平衡针) OR 题名或关键词 = 针)) AND (文摘 = 随机 OR 文摘 =RCT))

CBM
#1 " 偏头痛 "[不加权 : 扩展]
#2 " 原发性偏头痛 "[不加权 : 扩展]
#3 " 头痛 "[不加权 : 扩展]
#4 ((" 偏头痛 "[不加权 : 扩展]) OR (" 原发性偏头痛 "[不加权 : 扩展])) AND (" 头痛 "[不加权 : 扩展])
#5 " 偏头痛 "[常用字段 : 智能] OR " 原发性头痛 "[常用字段 : 智能] OR " 头痛 "[常用字段 : 智能]
#6 #4 OR #5
#7 " 针刺 "[不加权 : 扩展] OR " 针刺镇痛 "[不加权 : 扩展] OR " 针刺疗法 "[不加

权 ：扩展]
#8 " 针灸疗法 "[不加权 ：扩展]
#9 " 电针 "[不加权 ：扩展]
#10 " 头针 "[不加权 ：扩展]
#11 " 腕踝针 "[不加权 ：扩展]
#12 ("针刺"[常用字段:智能] OR "针灸"[常用字段:智能] OR "电针"[常用字段:智能] OR "头针"[常用字段:智能] OR "腕踝针"[常用字段:智能] OR "平衡针"[常用字段:智能] OR " 针 "[常用字段 ：智能]
#13 #7 OR #8 OR #9 OR #10 OR #11 OR #12
#14 " 随机对照试验 "[不加权 ：扩展]
#15 " 随机 "[常用字段 ：智能] OR "RCT"[常用字段 ：智能]
#16 #14 OR #15
#17 #6 AND #13 AND #16
